# Improving cell-type composition inference in spatial transcriptomics with SpaDAMA

**DOI:** 10.1371/journal.pcbi.1013354

**Published:** 2025-08-21

**Authors:** Lin Huang, Xiaofei Liu, Fangfang Zhu, Wenwen Min

**Affiliations:** 1 School of Information Science and Engineering, Yunnan University, Kunming, Yunnan, China; 2 School of Health and Nursing, Yunnan Open University, Kunming, Yunnan, China; University of Pittsburgh, UNITED STATES OF AMERICA

## Abstract

Accurate determination of cell-type composition in disease-relevant tissues is essential for identifying potential disease targets and understanding tissue heterogeneity. Most current spatial transcriptomics (ST) technologies lack single-cell resolution, which makes precise cell-type composition identification challenging. Several deconvolution methods have been developed to address this limitation by relying on single-cell RNA sequencing (scRNA-seq) data from the same tissue as a reference to estimate the cell type composition in ST data spots. However, these methods often overlook the inherent differences between scRNA-seq and ST data. To overcome this challenge, we introduce a Domain-Adversarial Masked Autoencoder (SpaDAMA) method. SpaDAMA leverages Domain-Adversarial Learning (DAL) to facilitate effective knowledge transfer from the source domain (pseudo-ST data generated from scRNA-seq) to the target domain (real ST data). Through adversarial training, SpaDAMA harmonizes the distributions of both datasets and maps them onto a unified latent representation, thereby reducing discrepancies in data modalities. Furthermore, to strengthen the model’s capability in extracting reliable features from real ST data, SpaDAMA employs masking strategies that effectively minimize noise and mitigate spatial artifacts. We validated SpaDAMA on 32 simulated datasets and 4 real-world datasets, demonstrating its superior performance in cell-type deconvolution and providing a promising tool for spatial transcriptomic analyses.

## Author summary

Spatial transcriptomics (ST) technology enables researchers to study the spatial distribution of gene expression in tissues, offering critical insights into cell interactions and disease mechanisms. However, most ST platforms lack single-cell resolution, as each spatial spot typically captures multiple cells. This limitation makes it difficult to accurately determine the cell-type composition at each location. To address this, scientists often integrate ST data with single-cell RNA sequencing (scRNA-seq) data for cell-type deconvolution. Yet, differences in data characteristics between ST and scRNA-seq limit the effectiveness of this approach. In this study, we introduce SpaDAMA, a novel deep learning framework that combines masked autoencoding with domain-adversarial learning. SpaDAMA effectively reduces the noise and spatial artifacts in ST data while aligning the distributions of real and simulated data to improve prediction accuracy. Through extensive evaluations on both simulated and real datasets, we demonstrate that SpaDAMA outperforms existing methods in both accuracy and robustness. Our method provides a powerful tool for resolving cell-type compositions in spatial transcriptomics, thereby enhancing our understanding of tissue architecture and supporting downstream biomedical research.

## 1. Introduction

Spatial transcriptomics (ST) data combine spatial context with gene expression patterns, offering invaluable insights into disease mechanisms and guiding targeted therapeutic strategies [1,2]. In cancer research, ST enables detailed mapping of gene expression within tumors, revealing tumor heterogeneity, microenvironment interactions, and potential biomarkers [3,4]. This technology also facilitates the creation of spatial maps of gene expression, highlighting spatial variations that are crucial for personalized treatment strategies [5]. Furthermore, ST data provide a deeper understanding of cell-cell interactions and cellular heterogeneity, shedding light on tissue architecture and function. Recent developments in ST emphasize leveraging this technology to investigate intricate biological systems and their interactions.

ST technologies can generally be grouped into sequencing-based and image-based approaches [6]. Image-based methods, such as seqFISH+ [7] and MERFISH [8], offer exceptional spatial resolution, allowing detection of gene expression at subcellular or even single-molecule levels. However, these techniques typically detect only a few genes [9]. Despite their high spatial precision, the few detectable genes restricts their ability to comprehensively profile the entire transcriptome in complex tissues. In contrast, Sequencing-based approaches, exemplified by the 10X Genomics Visium spatial transcriptomics platform [10], provide an alternative strategy. It has gained widespread adoption due to their ability to detect a broader range of genes, which typically encompasses thousands of transcripts. Although these methods Offer a broad perspective on gene expression across numerous genes, they generally offer lower spatial resolution compared to image-based techniques. This reduced resolution often results in each spatial spot containing multiple cells, blurring the distinction of gene expression signatures among different cell types [11]. Recent advancements, including spatially resolved RNA sequencing technologies like Slide-seq [12] and Stereo-seq [13], aim to improve spatial resolution while maintaining broad gene coverage. These approaches seek to combine the gene’s breadth of sequencing-based methods with higher spatial precision, enabling more detailed exploration of tissue architecture. However, achieving both high resolution and broad transcriptome coverage poses a considerable hurdle.

To overcome the limitations of single-cell resolution in ST data, several cell-type deconvolution algorithms have been proposed to estimate cellular composition at each spatial location [14,15]. Traditional approaches, such as SPOTlight [16] and CIBERSORT [17] , rely on scRNA-seq data as a benchmark profiles to infer the cellular makeup of ST spots. CIBERSORT, originally designed for bulk RNA-seq data, uses linear SVR to model the relationship between scRNA-seq profiles and ST signals, assuming that scRNA-seq data reflect aggregated gene expression patterns in ST data. Similarly, SPOTlight employs non-negative matrix factorization to partition ST data into cellular components, also assuming that scRNA-seq profiles are suitable references for this task. These methods often overlook critical differences between ST and scRNA-seq data, as scRNA-seq measures gene expression at the single-cell resolution, whereas ST data provide aggregated gene expression from multiple cells within a spatial spot. In addition, variations in sample preparation, spatial heterogeneity, and technical noise can further exacerbate discrepancies between these two data types [18]. These factors complicate the deconvolution process and can undermine the accuracy of cellular composition estimates. While more advanced methods, such as DestVI [19], integrate variational inference and deep learning to model cellular composition at spatial locations, they still encounter significant challenges in addressing the inherent discrepancies between scRNA-seq and ST data. Furthermore, Spoint [20] improves the alignment of real and synthetic ST data generated from scRNA-seq by employing a maximum mean discrepancy loss function. However, Spoint does not explicitly take into account the unique characteristics of real ST data during feature extraction, which may limit its ability to fully capture the underlying biological signals.

**Fig 1:**
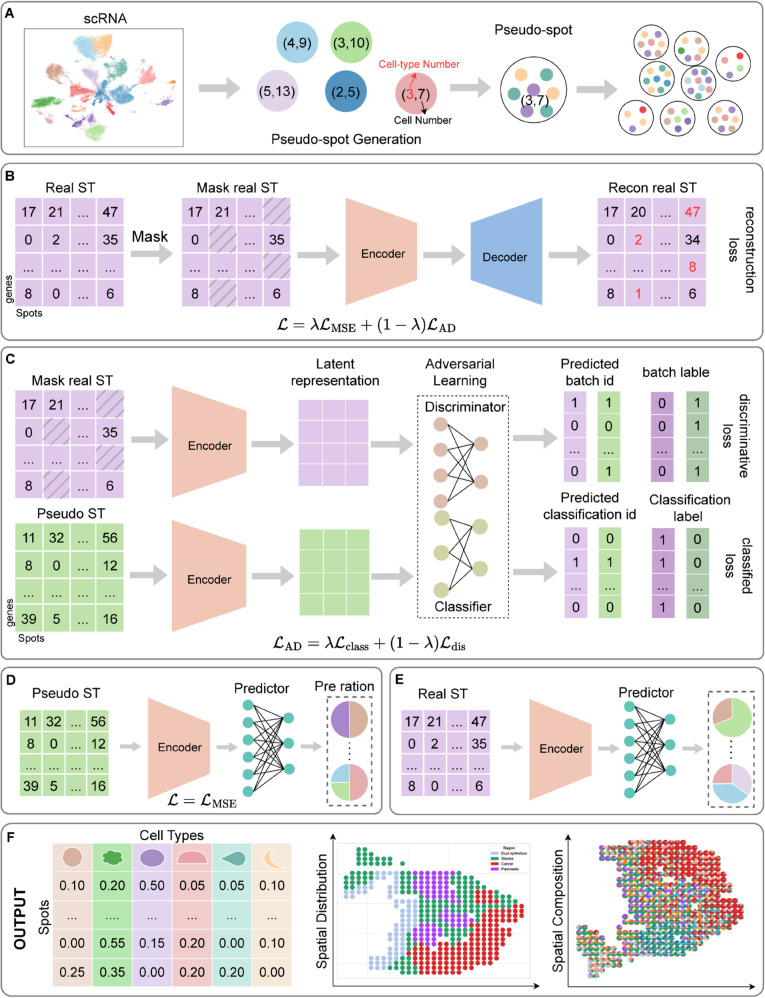
The network architecture of SpaDAMA. (**A**) Describes the procedure for pseudo-ST data generation. The SpaDAMA training phase consists of three stages: (**B**), (**C**), and (**D**). In these stages, the pseudo and masked ST data are processed by a shared Encoder, which generates latent variables using a masked autoencoder. (**E**) The optimized model is subsequently applied to estimate the cellular makeup of real ST data. (**F**) Downstream analysis.

To address these challenges, we propose SpaDAMA (Fig 1), a Domain-Adversarial Masked Autoencoder, designed to improve cell-type deconvolution in spatial transcriptomics. SpaDAMA utilizes a masked autoencoder [21] to uncover the intrinsic features of real ST data, optimizing SpaDAMA’s ability to learn true spatial expression patterns and improving generalization. The masked mechanism helps mitigate overfitting by forcing the model to learn robust features specific to real ST data, rather than over-relying on noisy or irrelevant signals. Recent extensions of masked autoencoders to structured data, such as GiGaMAE [22] and self-guided masking approaches [23], further support this strategy’s applicability in capturing biological signals. In addition, SpaDAMA generates pseudo-ST data from scRNA-seq data by randomly selecting cells, with known cell-type proportions. A domain-adaptation approach [24] is then applied to align simulated and real ST data in a shared embedding space. This approach assumes that gene expression profiles corresponding to identical cell types, whether derived from ST or scRNA-seq, should demonstrate high consistency [25]. Domain-adversarial strategies tailored for spatial integration, such as SPIRAL [26], also highlight the benefit of aligning cross-modal datasets in a unified representation space. By minimizing the discrepancies between real and simulated data, the domain adaptation process enhances SpaDAMA’s ability to generalize and ensures better consistency across data sources. Finally, SpaDAMA uses labeled pseudo-ST data to guide the training process, enabling it to accurately predict cellular composition in real ST data. As the model becomes proficient at distinguishing between real and simulated data, it is expected to perform well in predicting the true cellular composition of real ST data. To summarize, the coordinated exploration of ST and scRNA-seq data through SpaDAMA enables a deeper understanding of tissue architecture and spatial heterogeneity, providing insights into cellular interactions and disease mechanisms.

SpaDAMA demonstrates superior robustness and accuracy compared to nine existing methods, as validated on 32 simulated and 4 real-world datasets. These results highlight its ability to effectively handle the complexities inherent in ST data, offering a more reliable and comprehensive approach for cell-type deconvolution and providing deeper insights into tissue architecture and cellular interactions.

## 2. Results

### 2.1. Application to the 32 simulated datasets for benchmark evaluations

Assessing the accuracy of inferred cellular compositions in real ST data remains challenging due to the absence of single-cell granularity. To rigorously evaluate the performance of SpaDAMA, we utilized 32 synthetic datasets derived from a benchmark study [27]. These datasets were specifically constructed to emulate key features of real ST data, providing a dependable reference for assessing deconvolution accuracy.

**Fig 2:**
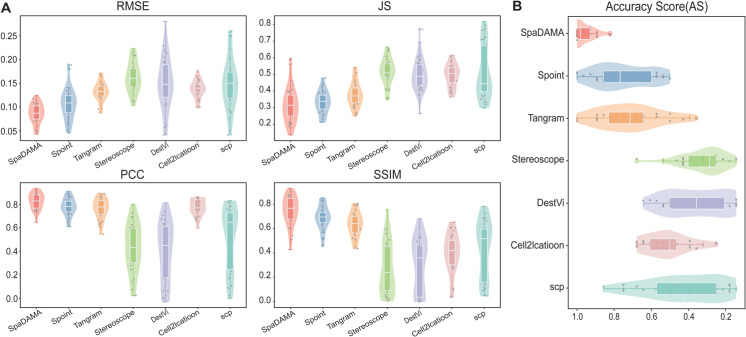
Performance evaluation of SpaDAMA across 32 simulated datasets demonstrated superior performance, as evidenced by higher PCC, SSIM, and AS values, along with lower RMSE and JS values. AS (Accuracy Score) is a composite metric that combines PCC, SSIM, RMSE, and JS.

We contrasted SpaDAMA with six advanced cell-type deconvolution methods to gauge its effectiveness. Since both SpaOTsc and NovoSpaRc require spatial location information for deconvolution and the pseudo-ST data lacks such positional data, these two methods were excluded from our evaluation. As a result, SpaDAMA was compared with six other deconvolution methods. Our experimental results demonstrated that SpaDAMA outperformed the others, achieving the highest mean PCC (0.937) and SSIM (0.930) scores and the lowest average RMSE (0.043) and JS (0.135) values (Fig 2A). Furthermore, when assessing overall performance using the Accuracy Score (AS), SpaDAMA achieved a significantly higher average AS (0.93) compared to other methods (AS = 0.32–0.75) (Fig 2B). Taken together, these results underscore the substantial advantages of SpaDAMA in cell-type deconvolution, highlighting its superior performance not only in similarity assessment but also in error measurement. Through extensive testing on simulated datasets, SpaDAMA has proven its robustness and exceptional capability in accurately predicting complex cell-type compositions.

### 2.2. SpaDAMA identifies cardiomyocytes in the human developing heart dataset

**Fig 3:**
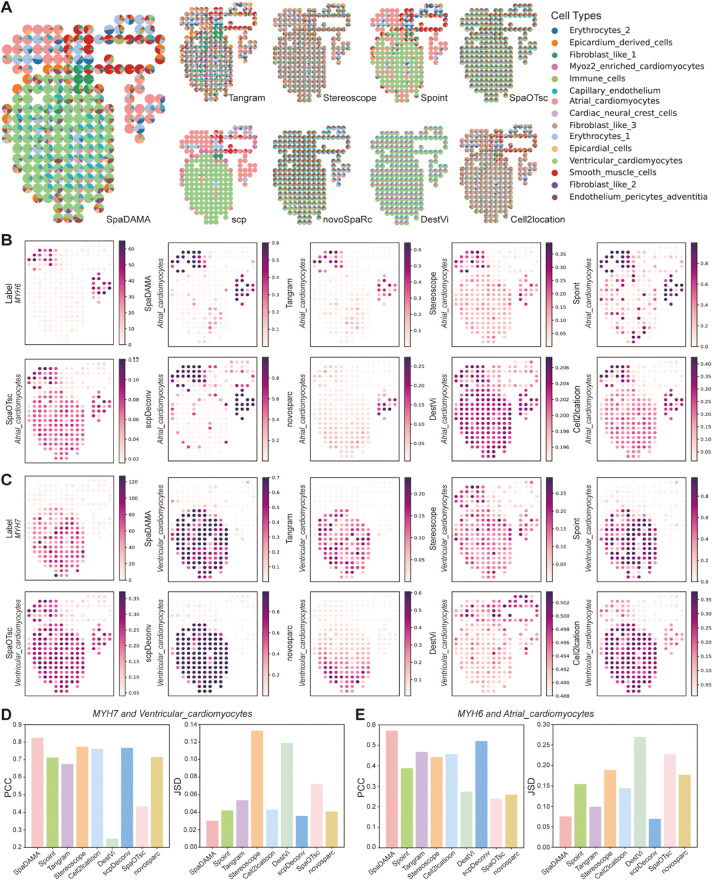
SpaDAMA accurately identifies the two subtypes of cardiomyocytes (atrial cardiomyocytes and ventricular cardiomyocytes) in the Human Developing Heart (HDH) dataset. (**A**) Compares the prediction results of various methods applied to the HDH dataset. (**B**) Displays the marker gene *MYH6* and the estimated proportions of atrial cardiomyocytes (a rare cell type), as determined by SpaDAMA and other methods. (**C**) Shows the marker gene *MYH7* and the estimated proportions of ventricular cardiomyocytes (a major cell type), as predicted by SpaDAMA and alternative methods. (**D**) Compares the performance of SpaDAMA and other methods in estimating the proportions of atrial cardiomyocytes, using PCC and JSD values for the marker gene *MYH6*. (**E**) Compares the performance of SpaDAMA and other methods in estimating the proportions of ventricular cardiomyocytes, using PCC and JSD values for the marker gene *MYH7*.

To assess SpaDAMA’s effectiveness on real tissue samples, we employed it to the human developing heart (HDH) dataset [28]. To provide additional insights, we also present the clustering results of the scRNA-seq profiles derived from the same tissues in the HDH dataset, as well as the selected marker genes corresponding to each cell type (S3 Fig). We first present the overall cell-type composition predictions from SpaDAMA and eight other methods (Fig 3A). The results demonstrated that SpaDAMA effectively captures cell-type proportions with high accuracy. For a more detailed evaluation, we examined atrial cardiomyocytes, identified using *MYH6* as a marker gene. These cells are essential for facilitating blood flow from the atria to the ventricles, ensuring proper cardiac function [29]. The spatial distribution predicted by SpaDAMA exhibited a strong correlation with the expression of *MYH6* (Fig 3B), attaining the lowest Jensen-Shannon Divergence (JSD) of 0.0657 and the highest Pearson correlation coefficient (PCC) of 0.5721 (Fig 3D). These findings underscored SpaDAMA’s capability in accurately resolving rare cell types within spatial transcriptomics data.

Subsequently, we analyzed the spatial localization of ventricular cardiomyocytes, using *MYH7* as a marker gene [30]. These cells reside in the ventricular chambers and play a pivotal role in systemic circulation. The predicted spatial distribution of ventricular cardiomyocytes closely aligned with the *MYH7* expression pattern (Fig 3C). In the quantitative analysis (Fig 3E), SpaDAMA achieved a PCC of 0.8242 and the lowest JSD of 0.0301, reinforcing its effectiveness in accurately reconstructing both the abundance and spatial organization of these cardiac cells.

In summary, our evaluation highlights SpaDAMA’s robustness in processing spatial transcriptomics data, particularly in scenarios involving spatially restricted or rare cell populations. The method demonstrates high fidelity in reconstructing cell-type distributions within the HDH dataset, offering a valuable tool for investigating human heart development at the cellular level. By reliably capturing the spatial organization of distinct cardiomyocyte subtypes, SpaDAMA provides deeper insights into their functional roles during cardiac maturation.

### 2.3. SpaDAMA identifies B cell subsets in the mouse lymph node dataset

**Fig 4:**
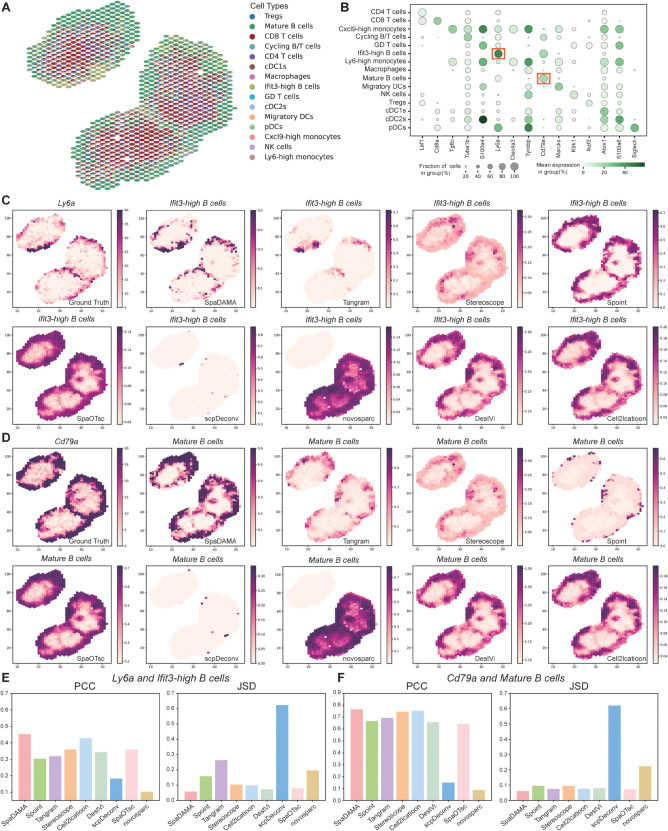
SpaDAMA effectively analyzes the cell composition in the Murine Lymph Node (MLN) dataset. (**A**) shows the cell composition results predicted by our method (SpaDAMA). (**B**) provides detailed information on the marker genes selected corresponding to each cell type. (**C**) Presents the marker gene *Ly6a* and the estimated proportions of Ifit3-high B cells, as predicted by SpaDAMA and other methods. (**D**) Shows the marker gene *Cd79a* and the estimated proportions of Mature B cells, as predicted by SpaDAMA and other methods. (**E**) Compares the PCC and JSD scores between SpaDAMA and other methods for the marker gene *Ly6a* and the estimated proportion of Ifit3-high B cells. (**F**) Compares the PCC and JSD scores between SpaDAMA and other methods for the marker gene *Cd79a* and the estimated proportion of Mature B cells.

To further evaluate SpaDAMA’s performance on different tissue environments, we utilized it to a mouse lymph node (MLN) dataset [19]. Differential expression analysis was first performed on the scRNA-seq data from the same tissue using the tools available in Scanpy [31], identifying marker genes corresponding to each cell type. As an example, *Ly6a* was selected as a marker gene for Ifit3-high B cells [32], while *Cd79a* was used as a marker for mature B cells [33]. By visualizing the spatial distribution of these two cell types and their associated marker gene expression (Fig 4B), we could clearly see that the cellular composition inferred by SpaDAMA accurately reflects the expected distribution of these cells (Fig 4C and 4D). Specifically, Ifit3-high B cells, which are typically associated with the early immune response to viral infections, are predominantly located in the marginal zone of the lymph node. In contrast, mature B cells, which play a crucial role in adaptive immunity, are mainly found in the superficial cortex. The results demonstrated that the positions predicted by SpaDAMA align well with the theoretical distribution, validating its high accuracy in cell type deconvolution (Fig 4C and 4D).

Next, the quantitative evaluation metrics show that SpaDAMA performs excellently in cell type deconvolution, achieving the highest PCC values (0.7645 and 0.4518, respectively), and the lowest JSD values (0.0606 and 0.0579, respectively), further confirming its superiority in precise deconvolution (Fig 4E and 4F). Finally, the overall prediction results from SpaDAMA showd high accuracy in cell type partitioning, effectively distinguishing the distribution of cells across different regions (Fig 4A).

### 2.4. SpaDAMA identifies lineage-specific cells in Zebrafish embryo dataset

**Fig 5:**
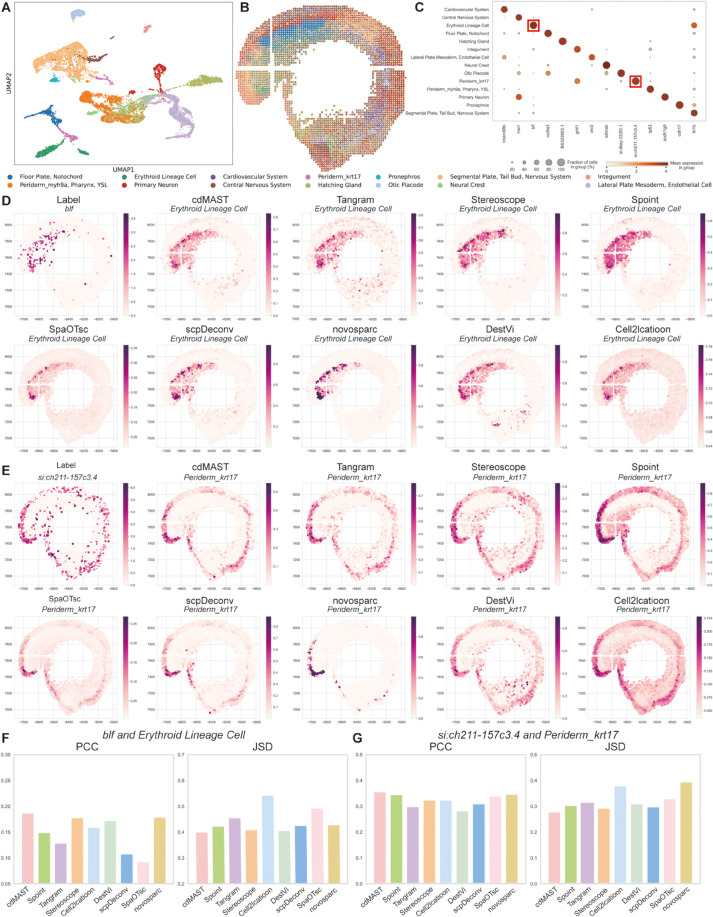
SpaDAMA shows strong performance in analyzing cell composition within the Zebrafish Embryo (ZE) dataset. (**A**) Clustering of scRNA-seq data by cell types from the same tissue. (**B**) Cell composition predicted by SpaDAMA. (**C**) Marker genes selected corresponding to each cell type. (**D**) Gene expression levels of *blf*, followed by predicted Erythroid Lineage Cell proportions by SpaDAMA and other methods. (**E**) Expression levels of *si:ch211-157c3.4*, followed by predicted Periderm_krt17 proportions by SpaDAMA and other methods. (**F**) PCC and JSD scores for *blf* and Erythroid Lineage Cell proportions. (**G**) PCC and JSD scores for *si:ch211-157c3.4* and Periderm_krt17 proportions.

To validate SpaDAMA’s applicability to different tissue types, we applied it to a Zebrafish Embryo (ZE) dataset [34]. Initially, cell type clustering analysis was performed on the scRNA-seq data derived from the same tissue (Fig 5A), which provided a foundation for subsequent analysis. Based on this analysis, detailed information on the marker genes selected for each cell type is presented (Fig 5C). For instance, the *blf* gene was chosen as a marker for Erythroid Lineage cells [35], which are typically concentrated in the dorsal region of the embryo during mid-development, specifically located at the upper part of the zebrafish embryo, where they play a critical role in blood circulation formation. Another cell type, Periderm_krt17 cells, was identified using the *si:ch211-157c3.4* gene [36]. These cells play a crucial role in shaping both the embryonic skin and nervous system, and are predominantly found in the surface region of the embryo, covering the outer layer and contributing to the formation of the epidermis and neural crest. Next, the predictions of these two cell types by SpaDAMA and other methods were compared (Fig 5D and 5E). Visualizing the spatial arrangement of these cells and their corresponding marker gene expression demonstrated that the predictions made by SpaDAMA align closely with theoretical expectations.

To further validate these results, quantitative evaluation metrics such as PCC and JSD were used to compare SpaDAMA’s performance with other methods (Fig 5F and 5G). The results indicated that SpaDAMA excelled in both metrics, confirming its superiority in cell type deconvolution. Finally, the overall prediction results from SpaDAMA showed highly accurate cell type partitioning, successfully distinguishing the distribution of cells across different regions (Fig 5B). These findings further corroborate the exceptional performance of SpaDAMA in the Zebrafish Embryo dataset, demonstrating its capacity to quantify the spatial distribution of diverse cell types.

### 2.5. Application to human pancreatic ductal adenocarcinoma dataset

**Fig 6:**
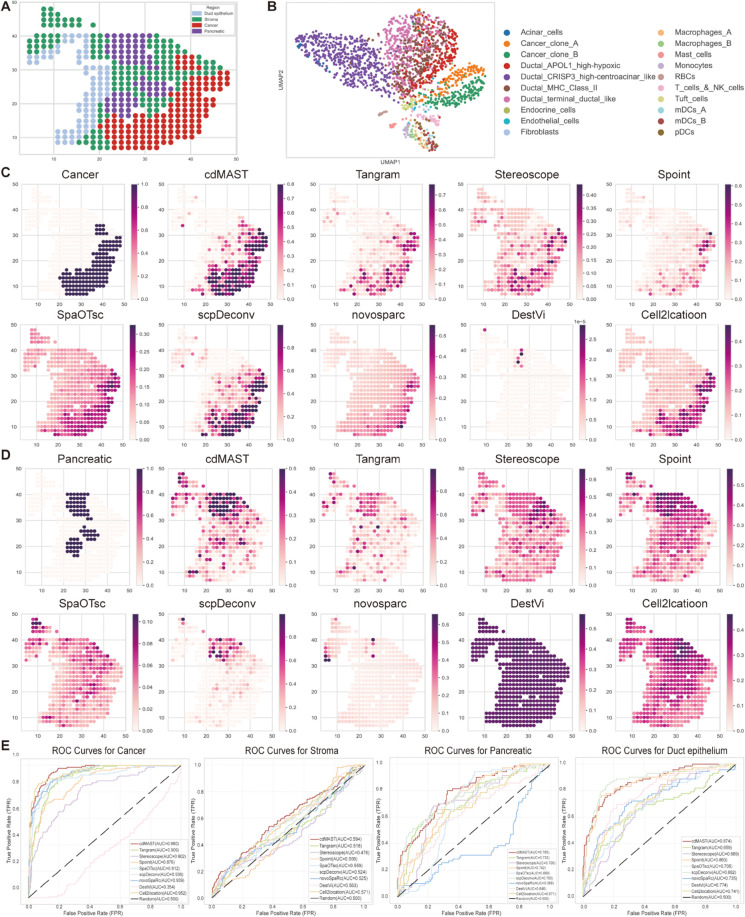
SpaDAMA demonstrates strong performance in analyzing cell composition within the Human Pancreatic Ductal Adenocarcinoma (PDAC) dataset. (**A**) shows the true labels for the four regions (cancer, stroma, pancreatic, and duct epithelium). (**B**) presents the clustering results of scRNA-seq data, grouped by cell types from the same tissue. (**C**) The first panel shows the cancer regions labeled, followed by panels displaying the prediction results from various methods. (**D**) The first panel shows the pancreatic regions labeled, followed by panels displaying the prediction results from various methods. (**E**) shows the ROC curves for the prediction of the four regions (cancer, stroma, pancreatic, and duct epithelium) by different methods.

Based on our previous analysis of three datasets, we extended evaluation to a human pancreatic ductal adenocarcinoma (PDAC) dataset [37] to further assess SpaDAMA’s performance. We first performed clustering analysis on the matching scRNA-seq data from the same patient (Fig 6B). The PDAC ST data includes four tissue regions: stroma, cancer, pancreas and duct epithelium, which were annotated by histopathologists based on H&E staining (Fig 6A) [38]. We referred to the study by Y. Ma [37] and explicitly presented the detailed distributions of specific cell types in S1 Table. Subsequently, SpaDAMA and other deconvolution methods localized pancreatic and tumor cell types to their corresponding tissue regions (Fig 6C and 6D). The visualization results showd that SpaDAMA effectively distinguishes cancerous regions from non-cancerous regions, with particular strength in distinguishing the pancreas region. In contrast, other methods fail to accurately partition these areas.

In the cancer region, which is primarily composed of cancer clones A and B, we visualized the cumulative predictions for these two cell types [39]. In the pancreas region, the main cell types include Acinar_cells, Endocrine_cells, and RBCs [40], and we similarly visualized the cumulative predictions for these cell types. Notably, since the region labels for each spot in the ST data are known, we plotted ROC curves for the four tissue regions and calculated the AUC values for each method (Fig 6E). The results show that SpaDAMA achieves the highest average AUC across all four regions, further confirming its robustness and superiority in cell type deconvolution.

### 2.6. Correlation analysis of predicted cell type proportions in spatial transcriptomics via SpaDAMA

**Fig 7:**
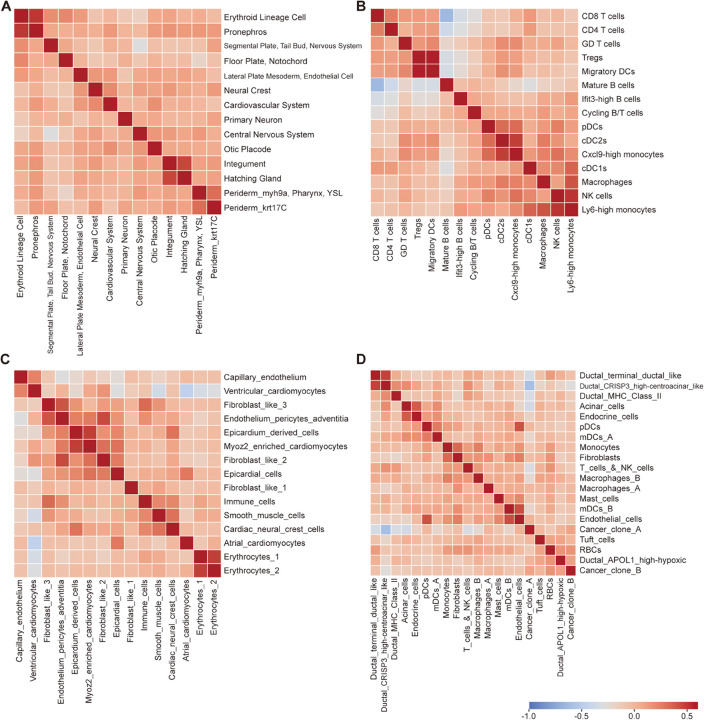
The heatmap displays the Pearson correlation scores of cell types, computed using the cell-type proportions inferred by SpaDAMA across four real ST datasets. The color scale represents the correlation values. (**A**) shows the Zebrafish Embryo (ZE) dataset, (**B**) shows the Mouse Lymph Node (MLN) dataset, (**C**) shows the Human Developing Heart (HDH) dataset, and (**D**) shows the Human Pancreatic Ductal Adenocarcinoma (PDAC) dataset.

To investigate the intercellular dynamics of key cell types across the four real ST datasets, we analyzed Pearson correlation scores [41,42] based on the deconvolution results inferred by SpaDAMA (Fig 7). In the zebrafish embryo dataset, certain cell types exhibit a strong correlation (Fig 7A). For instance, Erythroid Lineage Cells and Pronephros Cells both originate from the mesoderm during early embryonic development [43,44]. These two cell types are located in the lower regions of the embryo and are positioned adjacent to each other. Erythroid lineage cells contribute to early hematopoiesis, while pronephros cells are essential for the formation of the initial kidney structures. Their spatial proximity likely reflects the interaction and coordination of mesodermal tissues during embryogenesis. Additionally, Hatching Gland Cells are situated in the head region of the embryo, closely associated with the distribution of Integument Cells [45,46]. As the embryo develops, integument cells form a protective barrier on the surface, while hatching gland cells secrete enzymes to facilitate the hatching process. The spatial proximity and complementary functions of these two cell types suggest that they may coordinate and regulate each other’s activity during the hatching process [47].

The cell composition predicted by SpaDAMA clearly demonstrates the synergistic interactions between different cell types during immune responses in the mouse lymph node dataset (Fig 7B). For example, the interaction between the regulatory immune cells Tregs and Migratory DCs serve a key function in immune regulation [48]. Specifically, Tregs modulate the expression of co-stimulatory molecules on dendritic cells, thereby reducing their ability to activate effector T cells. This effectively modulates the intensity of immune responses, preventing excessive activation of the immune system. Meanwhile, Cxcl9-high monocytes and cDC2s play important roles in immune surveillance and antigen presentation [49]. During inflammatory responses, Cxcl9-high monocytes secrete the chemokine CXCL9, which recruits cDC2s to the site of inflammation, enhancing antigen presentation and T cell activation, thereby strengthening the immune response. Lastly, Ly6-high monocytes and NK cells collaborate in responding to pathogens and tumor cells. Ly6-high monocytes secrete cytokines such as IL-12 and IL-18, which promote the activation, proliferation, and cytotoxic function of NK cells. This interaction enables Ly6-high monocytes and NK cells to work closely together in antiviral and antitumor immune responses, effectively counteracting pathogen threats [50]. Through the finely tuned regulation and cooperation of these cells, the immune system maintains balance across different immune response scenarios, ensuring an adequate response to pathogens while preventing immune overactivation that could lead to tissue damage[51,52].

Our analysis of the human developing heart dataset reveals that SpaDAMA’s predictions uncover a strong correlation among certain cell types, highlighting their synergistic interactions during cardiac development (Fig 7C). For instance, Epicardium-derived cells secrete various growth factors (such as FGF, BMP) that regulate the proliferation, differentiation, and function of Myoz2_enriched_cardiomyocytes cells [53,54]. The interaction between these two cell types plays a crucial role in the early stages of heart development, driving angiogenesis and promoting the thickening of the heart wall [55,56]. Additionally, Endothelium_pericytes_adventitia cells and Fibroblast_like_2 cells collaborate closely in the process of vascular formation [57,58]. Endothelium_pericytes_adventitia cells primarily contribute to the development, stabilization, and upkeep of blood vessels, thus participating in the construction of the cardiac vasculature [55]. Meanwhile, Fibroblast_like_2 cells support these structures by synthesizing extracellular matrix components and plays an essential role in the structural and functional remodeling of the heart [58]. Moreover, Erythrocytes_1 cells and Erythrocytes_2 cells represent distinct erythrocyte subpopulations at different stages of cardiac development [59,60]. Erythrocytes_1 cells are early-stage erythrocytes in the embryo, primarily responsible for providing initial oxygen transport, while Erythrocytes_2 cells represent mature erythrocytes with adult-type hemoglobin, capable of more efficient oxygen transport. The gradual transition between these two erythrocyte subpopulations reflects adaptive changes in blood supply during heart development, ensuring that the heart meets its physiological demands at different developmental stages [59,60].

The predictions made by SpaDAMA in the human pancreatic ductal adenocarcinoma dataset provide insight into the cell interactions at various stages of tumor development, including early invasion, malignant transformation, and subsequent immune evasion mechanisms (Fig 7D). During the initial phases of PDAC, the crosstalk between Ductal_CRISP3_high−centroacinar_like and Ductal_terminal_ductal_like cells likely pivotal in the formation of the tumor microenvironment and its invasive characteristics [61]. Specifically, during Acinar-to-Ductal Metaplasia (ADM), Acinar_cells undergo transformation into ductal-like cells and interact with Endocrine_cells, driving tumorigenesis and progression [62]. This process is closely linked to early tumor initiation, proliferation, and local metastatic formation, potentially by modifying the composition of the tumor microenvironment, which fosters tumor malignancy. Moreover, Endothelial_cells and mDCs_B each play significant roles in immune surveillance, but in the PDAC tumor microenvironment, these cells’ functions may be suppressed by tumor cell-mediated immune evasion mechanisms [63]. Specifically, Endothelial_cells contribute to tumor angiogenesis by secreting immune-suppressive factors, such as VEGF, while potentially promoting immune tolerance to inhibit mDCs_B cells’ anti-tumor immune functions . This mechanism may diminish the ability of mDCs_B cells to activate effector T cells, thus allowing the tumor to evade immune surveillance and promoting immune escape [64].

### 2.7. Ablation studies

Details of the ablation studies of SpaDAMA are provided in the Sect 1.1 in S1 Text, S3 Table and S1 and S2 Figs.

## 3. Discussion

Spatial transcriptomics (ST) technology enables researchers to reveal the heterogeneity and spatial localization of cell types within complex tissues, thus advancing our understanding of tissue structure, disease progression, and cell-to-cell interactions. However, most existing ST techniques cannot achieve single-cell resolution and typically provide lower spatial resolution, which presents significant challenges in deconstructing the cellular composition of complex tissues. To address this challenge, we propose SpaDAMA, a model based on masked domain adversarial learning strategy, designed to learn shared spatial features across different data domains, thereby accurately inferring the cellular composition within tissue regions.

To effectively analyze transcriptomic data, methods such as Seurat [65] and SpaGE [66] aim to identify shared spatial features between ST and scRNA-seq data through joint embedding strategies, thus optimizing the multimodal data integration. Building on this concept, we hypothesize that there exist shared latent spatial features between pseudo-ST data generated from scRNA-seq data and real ST data from the same tissue. Based on this, SpaDAMA uses a domain adversarial mechanism to effectively reduce the distributional gap between the source domain (pseudo-ST data) and the target domain (real ST data), thereby strengthening the model’s generalization ability and improving its adaptability across different data sources. Additionally, SpaDAMA incorporates a masked autoencoder to learn the intrinsic features of the target domain, ensuring that the model retains the target domain’s features while extracting sufficient spatial features from the source domain to improve performance on the target task. Finally, SpaDAMA further optimizes model performance through supervised learning. After addressing the distribution discrepancy between target and source domains, the model’s capability to precisely estimate cell composition in the real dataset. By leveraging this integrated strategy, SpaDAMA not only enhances its adaptability to spatial transcriptomics data but also mitigates the impact of noise on model accuracy, ultimately achieving more precise cell type deconvolution.

**Fig 8:**
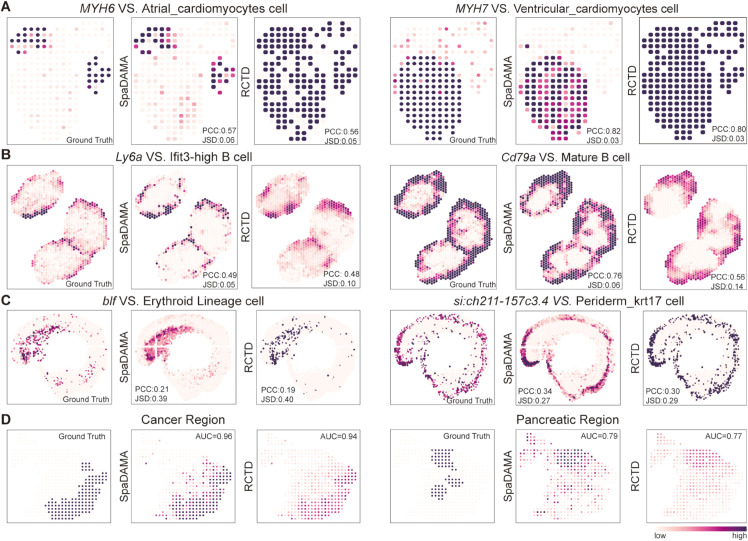
Comparison between SpaDAMA and RCTD on real spatial transcriptomics datasets. For each dataset, the figure presents the predicted spatial distributions of selected cell types, with corresponding marker genes indicated in italicized titles (*Marker Gene* VS. Cell Type). (**A**) Human Developing Heart (HDH) dataset. (**B**) Murine Lymph Node (MLN) dataset. (**C**) Zebrafish Embryo (ZE) dataset. (**D**) Human Pancreatic Ductal Adenocarcinoma (PDAC) dataset.

In the 32 simulated datasets from the benchmark experiments [27], we conducted a quantitative evaluation of SpaDAMA. The results demonstrated that the model exhibited outstanding accuracy in predicting the cell mixture and actual cell composition, with an average accuracy score of 0.93, confirming its high performance across different datasets. Subsequently, we applied SpaDAMA to evaluate 4 real datasets. The results showed that the model could accurately identify the cellular composition in different tissue regions, and the predicted cell type distribution was generally consistent with the theoretically expected regional distribution. Quantitative assessments also indicated superior performance.To further validate the effectiveness of SpaDAMA, we compared it with RCTD, a representative Bayesian-based deconvolution method. While Cell2location and Stereoscope also adopt Bayesian frameworks, RCTD distinguishes itself by explicitly incorporating spatial location information, which enhances the precision of cell composition estimation. We present a direct comparison between SpaDAMA and RCTD across multiple real datasets (Fig 8). The results demonstrate that SpaDAMA slightly outperforms RCTD in prediction accuracy, further confirming its robust generalization capability. These findings suggest that SpaDAMA not only demonstrates excellent predictive capability on simulated data but also maintains high accuracy on real data, further validating its broad applicability and reliability.

However, despite SpaDAMA’s strong performance across various datasets, the spatial resolution and data quality of ST technology remain critical factors affecting deconvolution accuracy. To address this, future work will focus on optimizing the masking strategy by incorporating spatial graph structures or histological image features, enabling more fine-grained and biologically informed masking designs. In addition, integrating higher-resolution ST data or other auxiliary information is expected to improve SpaDAMA’s adaptability and prediction accuracy for more complex tissue contexts. As ST technology continues to evolve, we also plan to extend SpaDAMA to support integrative analysis of multimodal spatial omics data, such as spatial epigenomics and spatial proteomics, thereby further enhancing its potential in deciphering complex tissue microenvironments and advancing biological research.

In conclusion, SpaDAMA provides an innovative solution to the problem of cell type deconvolution. By combining masked autoencoders and domain adversarial learning strategies, it effectively addresses the resolution limitations of ST data. Compared to traditional methods, SpaDAMA enables efficient cross-domain learning across multiple datasets and demonstrates excellent predictive capability in both simulated and real data. As a powerful tool, SpaDAMA not only offers new approaches for precise cell type prediction and in-depth tissue structure analysis but also paves the way for future broad applications in fields such as oncology, immunology, and developmental biology.

## 4. Methods

### 4.1. Dataset description

We leveraged 32 benchmark-simulated datasets [27] derived from seven distinct human and mouse tissue types (S2 Table). These datasets served as a foundation for quantitatively assessing the performance of our model.

Furthermore, we incorporated four real ST datasets spanning different biological systems: the human developing heart (HDH) [28], mouse lymph node (MLN) [19], zebrafish embryo (ZE) [34], and human pancreatic ductal adenocarcinoma (PDAC) [37] (Table 1). Specifically, the HDH dataset includes 210 spatial spots, accompanied by scRNA-seq data comprising 3,777 cells classified into 15 distinct cell types. The MLN dataset contains 1,092 spatially resolved spots, with scRNA-seq data encompassing 14,989 cells assigned to 15 unique cell types. For the ZE dataset, the Stereo-seq-based ST data capture 3,048 spatial spots, mapping 9,903 cells across 14 defined cell types. Lastly, the PDAC dataset consists of 426 spatial locations, corresponding to scRNA-seq data from 1,926 single cells representing 20 diverse cell types.

**Table 1:** Summary of real datasets used for analysis.

Dataset	Tissue	SC (Cells × Genes)	ST (Spots × Genes)
HDH	Human Developing Heart [28]	3777 × 15323	210 × 38936
MLN	Mouse Lymph Node [19]	14989 × 11740	1092 × 11740
ZE	Zebrafish Embryo [34]	9903 × 23986	3048 × 23110
PDAC	Pancreatic Ductal Adenocarcinoma [37]	1926 × 14121	426 × 14121

These datasets collectively provide a comprehensive foundation for evaluating the model’s ability to resolve cell-type distributions in varying biological contexts.

### 4.2. Data preprocessing

We utilized several tools, including Scanpy [31] and Seurat [65], to perform cell clustering on the scRNA-seq data and to identify marker genes corresponding to each cell type. From these marker genes, we selected the top 200 genes [67,68] for each cell type to ensure that the most representative features were captured for downstream analysis. For ST data, we focused on the genes that were common between the ST data and preprocessed scRNA-seq data, retaining only the intersecting genes to ensure consistency, for more details, please refer to S2 Table.

### 4.3. Mechanism of pseudo-ST data generation

Pseudo-spots are generated from scRNA-seq data, with their cellular composition inferred based on the included cell types. Specifically, we assume that the number of cells per spot (*N*_*t*_) and the number of cell types (*N*_*c*_) follow a normal distribution. First, we randomly select the total number of cells *t* from *N*_*t*_, and then randomly choose the number of cell types *c* from *N*_*c*_. Next, we generate a random sequence of numbers that sum to *t* based on the selected *c* cell types. For each selected cell type *c*_*i*_, we randomly sample *t*_*i*_ cells, which together form the pseudo ST data. During the sampling procedure, we tally the distribution of cell types among the sampled cells, compute the percentage of each cell type, and use this information as the label for each pseudo-spot (See Fig 1A). The detailed parameter settings used for simulating pseudo-spots are provided in the Sect 1.3 in S1 Text and S4 Table.

### 4.4. Masked autoencoder learning of SpaDAMA

Using a random masking strategy with a masking rate of *ρ*, we apply it to the real ST data matrix (*X*_*r*_) to generate a new masked data matrix ($X_{r}^{m}$) (see Fig 1B).

**Encoder** ℰ(·) processes the masked ST data by passing it through two stacked fully connected layers to produce a latent representation of gene expression:

$$H_{R}=ℰ(X_{r}^{m};\Theta_{e})=W_{e}^{1}\phi (\text{BN}(W_{e}^{0}X^{m}+b_{e}^{0}))+b_{e}^{1}$$ (1)

where $W_{e}^{0}$ and $W_{e}^{1}$ are the weight matrices, $b_{e}^{0}$ and $b_{e}^{1}$ are the bias terms, BN denotes batch normalization, and ϕ represents the LeakyReLU activation function.

**Decoder** 𝒟(·) consists of three fully connected layers that are used to reconstruct gene expression based on the latent representation:

$${\hat{X}}_{r}=𝒟(H_{R};\Theta_{d})$$ (2)

Similar to the Encoder, the Decoder consists of fully connected layers with corresponding weight matrices and bias terms, although it uses three layers instead of two. Since the structure of the Decoder mirrors that of the Encoder, it is not elaborated further here.

$${ℒ}_{\text{MSE}}=\|({\hat{X}}_{r}-X_{r})⊙M{\|}_{2}^{2}$$ (3)

where ⊙ denotes element-wise multiplication, and *M*_*ij*_ is either 1 or 0. A value of 1 indicates the element is masked, and 0 indicates it is non-masked.

### 4.5. Adversarial learning of SpaDAMA

The encoder processes masked real ST data and pseudo-ST data, generating their corresponding latent representations (Fig 1C). The first half of each latent representation is fed into a Feedforward Neural Network (FNN) **Classifier** $F_{C}(\cdot )$ to produce predicted labels. The model is optimized to effectively distinguish between real and pseudo data:

$$O_{C,R}=F_{C}(H_{R_{1}};\Theta_{C}),O_{C,S}=F_{C}(H_{S_{1}};\Theta_{C})$$ (4)

where $H_{R_{1}}$ and $H_{S_{1}}$ are the first halves of the features from *H*_*R*_ and *H*_*S*_, respectively. $H_{S}=ℰ(X_{S};\Theta_{e})$, with *X*_*S*_ denoting the pseudo ST data. $\Theta_{C}$ represents the classifier’s weights and biases. The loss of Classifier:

$${ℒ}_{\text{C}}=f({𝐎}_{C,R},{𝐎}_{1,T})+f({𝐎}_{C,S},{𝐎}_{0,T})$$ (5)

where ${𝐎}_{i,T}=[i,\ldots ,i],\text{ for }i\in \{0,1\}$, label *i* = 1 indicates real ST data and *i* = 0 indicates pseudo ST data. And the binary cross entropy (BCE) function is formulated as

$$f(y_{i},{\hat{y}}_{i})=-\frac{1}{N}\sum_{i=1}^{N}\left[y_{i}\log({\hat{y}}_{i})+(1-y_{i})\log(1-{\hat{y}}_{i})\right]$$ (6)

Next, the remaining features are fed into a **Discriminator** $F_{D}(\cdot )$, implemented as FNN. The discriminator is trained with incorrect labels, prompting it to misclassify data as real or pseudo in the latent space:

$$O_{D,R}=F_{D}(H_{R_{2}};\Theta_{D}),O_{D,S}=F_{D}(H_{S_{2}};\Theta_{D})$$ (7)

where *H*_*R*2_ and *H*_*S*2_ represent the remaining features of *H*_*R*_ and *H*_*S*_, respectively. $\Theta_{D}$ represents the discriminator’s weights and biases. The loss of Discriminator:

$${ℒ}_{\text{D}}=f({𝐎}_{D,R},{𝐎}_{0,T})+f({𝐎}_{D,S},{𝐎}_{1,T})$$ (8)

To validate the design choice of splitting latent features equally between the classifier and discriminator branches, we conducted ablation experiments on four real ST datasets by varying the allocation ratios 1:3, 1:1, and 3:1 (i.e., a 1:3 ratio indicates that 1/4 of the latent features are allocated to the classifier and 3/4 to the discriminator). The results, visualized in S4 Fig, demonstrate that the 1:1 allocation consistently achieves superior performance in terms of accuracy and training efficiency. Hence, we adopted the 1:1 ratio in this study.

### 4.6. Supervised learning of SpaDAMA

Define the supervised learning module as **Predictor** 𝒫(·) (Fig 1D). It is composed of two linear layers, followed by a softmax layer, which are specifically designed to infer cell-type proportions from the latent representation:

$$Y=𝒫(H_{S};\Theta_{P})=\text{softmax}(W_{p}^{1}\phi (\text{BN}(W_{p}^{0}H_{S}+b_{p}^{0}))+b_{p}^{1})$$ (9)

where $W_{p}^{0}$ and $W_{p}^{1}$ are the weight matrices, $b_{p}^{0}$ and $b_{p}^{1}$ are the bias terms. The loss function of the Predictor:

$${ℒ}_{P}=\|Y-Y_{r}{\|}_{2}^{2}$$ (10)

where *Y*_*r*_ refers to the ground truth cellular proportion of the pseudo ST data.

### 4.7. Training process of SpaDAMA

SpaDAMA applies domain adaptation techniques to infer the cellular makeup of real ST samples. Specifically, this approach effectively narrows the gap between pseudo-ST data (source domain) and real ST data (target domain), facilitating encoder generalization and ensuring greater alignment in data distribution. The domain adaptation process consists of two key components: supervised learning on the source domain and adversarial learning. We will detail the training process as follows.

To facilitate transfer learning in SpaDAMA through the adversarially supervised domain adaptation strategy, we alternate between the supervised learning component and the adversarial learning component. In the loss calculation process, each training iteration is divided into three sub-steps.

(1) The first sub-step is the supervised learning of the data, where the Masked Autoencoder (MAE) is trained with a loss function, while the other structures remain inactive. The optimization process can be expressed as:

$${ℒ}_{stage1}=\lambda {ℒ}_{\text{MSE}}+(1-\lambda ){ℒ}_{\text{BCE}}$$ (11)

$$\min_{\Theta_{e},\Theta_{d}}L_{stag_{1}}=ℱ\left(ℰ,𝒟\right)$$ (12)

where ${ℒ}_{\text{BCE}}={ℒ}_{\text{C}}\;+\;{ℒ}_{\text{D}}$ (See Eqs 5 and 8) and *λ* serves as a weighting factor to regulate the impact of reconstruction loss ${ℒ}_{\text{MSE}}$ and ${ℒ}_{\text{BCE}}$. ℱ indicates the activated modules in this sub-stage.

(2) In the second sub-step, adversarial training is applied to the classifier and discriminator using both pseudo and real ST data. During this process, the adversarial signal generated by the classifier’s correct predictions and the discriminator’s incorrect predictions is used to guide model optimization. Except for the discriminator and classifier, the other components remain unchanged during this step:

$${ℒ}_{\text{stage2}}=\lambda {ℒ}_{\text{C}}+(1-\lambda ){ℒ}_{\text{D}}$$ (13)

$$\min_{\Theta_{C},\Theta_{D}}L_{stag_{2}}=ℱ\left(F_{C},F_{D}\right)$$ (14)

where *λ* controls the trade-off between different components of the reconstruction loss ${ℒ}_{\text{C}}$ and ${ℒ}_{\text{D}}$.

(3) The third sub-step involves supervised learning using pseudo ST data to optimize the encoder and predictor. The optimization process can be expressed as:

$${ℒ}_{stage3}={ℒ}_{P}$$ (15)

$$\min_{\Theta_{e},\Theta_{P}}L_{stag_{3}}=ℱ\left(ℰ,𝒫\right)$$ (16)

To assess the stability of adversarial training, we monitored the loss dynamics during optimization. As shown in S5 Fig, the adversarial loss converges smoothly without oscillations across four real ST datasets. The observed patterns are consistent with scpDeconv [69], indicating that our adversarial training procedure is stable and reliable.

### 4.8. Inference of SpaDAMA

After repeated training, all parameters in SpaDAMA are fully optimized. Ultimately, SpaDAMA acquires knowledge of cell type composition from the source domain (pseudo ST data). At the same time, the domain adaptation strategy enables SpaDAMA to transfer this domain-specific knowledge and apply it to predict cell type composition in the target domain (real ST data) (Fig 1E):

$$Y_{t}=𝒫(ℰ(X_{r};\Theta_{e});\Theta_{P})$$ (17)

where *Y*_*t*_ denotes the final predicted cellular proportions for the real ST data.

### 4.9. Baselines

To assess the performance of SpaDAMA, we compared it with nine other state-of-the-art methods. The methods compared include the following:

- Tangram [70]: Tangram utilizes deep learning for aligning ST with scRNA-seq data. By training a deep neural network model, it predicts the spatial distribution of cell types, thereby performing the deconvolution task.The mapping of cells to space was conducted with the function tg.map_cell_to_space with mode =clusters.
- Stereoscope [71]: Stereoscope combines scRNA-seq and ST data, using a Bayesian inference model to probabilistically estimate cell type composition at spatial locations, enabling accurate cell type deconvolution. The method was run with parameters set as follows: n_top_genes = 7000, ST epochs = 300, SC epochs = 500, and learning rate = 0.01.
- Spoint [20]: By leveraging deep learning models to extract features and applying PCA for reducing feature dimensions, Spoint enhances the accuracy of cell-type deconvolution. The parameters were set as follows: sm_size = 10000, max_steps = 3000, sm_lr = 0.01, and st_lr = 0.01.
- SpaOTsc [72]: SpaOTsc leverages optimal transport theory to match single-cell RNA-seq data with spatial transcriptomics (ST) data for accurate cell-type deconvolution. The parameter transport_plan was set to cost_matrix.
- scpDeconv [69]: scpDeconv employs a domain-adversarial deep learning model for cell type deconvolution. It aligns data from different sources using an adversarial learning framework, mitigating data biases, and accurately estimates cell type proportions from tissue proteome profiling data. The model was trained with num_epochs = 200 and batch_size = 100.
- NovoSpaRc [73]: NovoSpaRc also employs optimal transport to link single-cell RNA-seq data and spatial gene expression data, but it focuses on reconstructing the spatial distribution of cell types across the tissue. Alpha was set as 0.5.
- DestVI [19]: DestVI uses variational inference and hidden factor models to estimate cell-type distributions, capturing their continuum and spatial heterogeneity for accurate deconvolution. DestVI filters genes with fewer than 10 counts using sc.pp.filter_genes. The single-cell model is trained for 300 epochs, and the spatial model for 1200 epochs with a learning rate of 10^−3^.
- Cell2location [74]: Cell2location uses a hierarchical Bayesian framework to estimate cell type proportions at each spatial location by integrating scRNA-seq and ST data, accounting for data uncertainty to achieve accurate deconvolution. The settings max_epochs = 2500, batch_size = None, and train_size = 1 were used.
- RCTD [75]: RCTD utilizes single-cell transcriptomic data as a reference and employs a Bayesian framework to robustly estimate the cell type composition at each spatial location. Spacexr (RCTD) was run with following the configuration: (1) create:RCTD was used with the parameter CELL_MIN_INSTANCE = 1; (2) run: RCTD was used in the doublet mode.

### 4.10. Evaluation metrics

We use structural similarity index measure (SSIM), Pearson correlation coefficient (PCC), Jensen-Shannon divergence (JS/JSD), root mean square error (RMSE), Accuracy score (AS) and Area Under the Receiver Operating Characteristic Curve (AUC) to evaluate the proposed method against baselines. Their definitions are as follows:

$$\text{PCC}=\frac{\text{cov}(x_{i},{\hat{x}}_{i})}{\sigma_{i}\hat{\sigma_{i}}}$$ (18)

Where *x*_*i*_ represents the ground truth composition of cell type *i*, $\sigma_{i}$ denotes its standard deviation, and ${\hat{x}}_{i}$ and $\hat{\sigma_{i}}$ are the corresponding predicted values.

$$\text{SSIM}=\frac{\left(2\hat{u_{i}}u_{i}+C_{1})(2\text{cov}(x_{i},{\hat{x}}_{i})+C_{2}\right)}{\left({\hat{u_{i}}}^{2}+u_{i}^{2}+C_{1})({\hat{\sigma_{i}}}^{2}+\sigma_{i}^{2}+C_{2}\right)}$$ (19)

Where $\mu_{i}$ denotes the average ground truth composition of cell type *i*, ${\hat{\mu }}_{i}$ is the predicted average, and *C*_1_ and *C*_2_ are constants, set to 0.01 and 0.03, respectively.

$$\text{RMSE}=\sqrt{\frac{1}{M}\sum_{j=1}^{M}(x_{ij}-\hat{x_{ij}}{)}^{2}}$$ (20)

where *x*_*ij*_ is the ground truth distribution of cell types *i* in spot *j*, and ${\hat{x}}_{ij}$ is the predicted value.

$$\text{JS}=\frac{1}{2}\text{KL}\left(P_{i},\frac{\hat{P_{i}}+P_{i}}{2}\right)+\frac{1}{2}\text{KL}\left(\hat{P_{i}},\frac{\hat{P_{i}}+P_{i}}{2}\right)$$ (21)

where *P*_*i*_ and ${\hat{P}}_{i}$ are the spatial distributions of cell type *i* in the ground truth and prediction, respectively.

$$\text{AS}=\frac{1}{4}({\text{RANK}}_{\text{SSIM}}+{\text{RANK}}_{\text{PCC}}+{\text{RANK}}_{\text{RMSE}}+{\text{RANK}}_{\text{JS}})$$ (22)

where the deconvolution methods are ranked by their average PCC/SSIM in ascending order and by their JS/RMSE in descending order, to obtain ${\text{RANK}}_{\text{SSIM}}$, ${\text{RANK}}_{\text{PCC}}$, ${\text{RANK}}_{\text{RMSE}}$, and ${\text{RANK}}_{\text{JS}}$.

$$\text{AUC}=\int_{0}^{1}\text{TPR}(t)\;d\text{FPR}(t)$$ (23)

where TPR(t) is the True Positive Rate and FPR(t) is the False Positive Rate at a given threshold *t*. The area under the curve (AUC) measures the model’s performance in distinguishing between positive and negative classes.

### 4.11. Cell type correlation score

To quantify the relationship between the abundances of two different cell types inferred by SpaDAMA across all sampled points, we compute the Pearson correlation coefficient to assess their linear association. Given a cell abundance matrix 𝐀, where *A*_*m*,*i*_ represents the abundance of cell type *i* at sample *m*, the correlation coefficient between cell types *i* and *j* is defined as:

$$r_{i,j}=\frac{\sum_{m=1}^{M}(A_{m,i}-{\bar{A}}_{i})(A_{m,j}-{\bar{A}}_{j})}{\sqrt{\sum_{m=1}^{M}(A_{m,i}-{\bar{A}}_{i}{)}^{2}}\sqrt{\sum_{m=1}^{M}(A_{m,j}-{\bar{A}}_{j}{)}^{2}}}$$ (24)

where *A*_*m*,*i*_ and *A*_*m*,*j*_ are the abundances of cell types *i* and *j* at sample *m*, ${\bar{A}}_{i}$ and ${\bar{A}}_{j}$ are the mean abundances of cell types *i* and *j* across all samples, *M* is the total number of samples.

### 4.12. Experiment settings

All baseline models were implemented using the default parameters from their original papers. The experiments were performed on an NVIDIA RTX 3090 GPU with PyTorch (version 1.12.1) and Python 3.9. The training was conducted over 200 epochs, with a batch size of 2048, a learning rate of 0.01, *ρ* = 0.3 and *λ*=0.5. Additionally, we recorded the runtime and memory usage of SpaDAMA and other methods across four real datasets, and the results are presented in Sect 1.2 in S1 Text and S6 Fig.

## Supporting information

S1 Text Supplementary notes for SpaDAMA. (1.1) Ablation studies. (1.2) Runtime and Memory Usage. (1.3) Simulation Parameter Settings for Spatial Transcriptomics Data.
(PDF)

S1 Fig Performance evaluation of SpaDAMA at different mask rates *ρ* across 32 simulated datasets. Metrics include Pearson correlation coefficient (PCC), Structural Similarity Index Measure (SSIM), Root Mean Square Error (RMSE), and Jensen-Shannon (JS) divergence.
(PDF)

S2 Fig Ablation study results on four real spatial transcriptomics datasets. Datasets 1 to 3 are evaluated with Pearson correlation coefficient (PCC), and Dataset 4 is evaluated with Area Under the Curve (AUC).
(PDF)

S3 Fig Cell type analysis in the Human Developing Heart (HDH) dataset. (A) UMAP clustering plot showing distinct scRNA-seq cell type clusters. (B) Marker genes selected for each cell type, highlighting characteristic expression patterns.
(PDF)

S4 Fig Impact of varying latent feature allocation ratios between classifier and discriminator on SpaDAMA’s performance across four real ST datasets: HDH, Murine Lymph Node (MLN), Zebrafish Embryo (ZE), and Human Pancreatic Ductal Adenocarcinoma (PDAC). (PDF)

S5 Fig SpaDAMA loss curves for four real datasets. Includes total loss, prediction loss, reconstruction loss on masked input, classification loss, and discrimination losses under correct and label-inverted conditions.
(PDF)

S6 Fig Runtime and peak memory usage comparisons of nine deconvolution methods on four real-world spatial transcriptomics datasets of varying sizes. (PDF)

S1 Table Cell type distributions by tissue region in the PDAC dataset, listing cell types enriched in Duct epithelium, Cancer, Pancreatic, and Stroma regions. (PDF)

S2 Table Detailed information on all datasets used in the study. Includes tissue type, data types (scRNA-seq and ST), cell and spot counts, gene counts, and dropout rates for source and preprocessed data.
(PDF)

S3 Table Ablation experiment results on 32 simulated datasets measured by the AS metric. Comparisons among baseline models with and without masking and adversarial learning, and full SpaDAMA model.
(PDF)

S4 Table Recommended parameter settings for simulating the number of cells and cell types per spot across different ST technologies. (PDF)
