# Supplementary material for: Improving cell-type composition inference in spatial transcriptomics with SpaDAMA
Source: PLoS Comput Biol. 2025 Aug 21;21(8):e1013354. doi: 10.1371/journal.pcbi.1013354 (PMC12393736; doi:10.1371/journal.pcbi.1013354)
Supplement: S1 Text — (1.1) Ablation studies. (1.2) Runtime and Memory Usage. (1.3) Simulation Parameter Settings for Spatial Transcriptomics Data. (PDF) [file pcbi.1013354.s001.pdf]

Supplementary Materials for

Improving Cell-Type Composition Inference in Spatial Transcriptomics with SpaDAMA

Supplementary Notes

1.1 Ablation studies

To evaluate the contributions of the masking and adversarial modules to the SpaDAMA model, we conducted a series of ablation experiments (S1 and S2 Fig and S3 Table). We specifically aimed to determine if these modules enhance model performance and whether reconstruction loss should be computed on the masked region or the entire ST data, as well as the impact of different masking rates. The experiments included a baseline model with only the encoder and predictor, a model with a masking module (reconstruction loss computed only for the masked region), a model with full reconstruction loss, and a model with an adversarial module, with masking rates $\rho = 0.2$, 0.3, 0.4, and 0.5. The ablation experiments confirm the critical role of the masking and adversarial modules in enhancing the accuracy of cell type proportion predictions, with the impact of the masking rate on the model's performance being relatively minor. In particular, the model performed best when the masking rate was $\rho = 0.3$.

Additionally, we explored the effect of the hyperparameter λ, which balances the contributions of the supervised and adversarial objectives in the total loss function. We tested three different values: $\lambda = 0.1$, $\lambda = 0.5$, and $\lambda = 0.9$. The average Accuracy Score (AS) across the 32 simulated datasets was 0.61, 0.75, and 0.63, respectively. These results suggest that $\lambda = 0.5$ provides the best trade-off, and we therefore adopted it in our final model.

1.2 Runtime and Memory Usage

As shown in S6 Fig, SpaDAMA demonstrates stable and efficient runtime performance, especially on larger datasets, where it outperforms several computationally intensive methods such as Spoint and Destivi. At the same time, SpaDAMA maintains reasonable memory usage, reflecting efficient resource management.Compared to some faster methods like Tangram, SpaDAMA's runtime is moderately longer, but it achieves higher accuracy and stability. Considering runtime, memory consumption, and prediction accuracy together, SpaDAMA exhibits excellent scalability and practical applicability across diverse real-world datasets.

1.3 Simulation Parameter Settings for Spatial Transcriptomics Data

Based on experimental knowledge and existing methods such as **scDesign3** [1] and **SPACEL** [2], we set simulation parameters reflecting typical cell counts per spot: 3–10 cells for Visium, 1–2 cells for Slide-seq, and near single-cell resolution for MERFISH. The number of cells per spot (N_c) and the number of cell types per spot (N_t) are modeled as normal distributions with means and standard deviations $\mu_c = 10, \delta_c = 5$ and $\mu_t = 5, \delta_t = 2.5$, respectively. These settings better capture the biological complexity and heterogeneity of spatial transcriptomics datasets. We also propose table (S4 Table) of parameters as a reference for different spatial transcriptomics technologies.

References

1. D. Song, Q. Wang, G. Yan, et al. scdesign3 generates realistic in silico data for multimodal single-cell and spatial omics. *Nature Biotechnology*, 42:247–252, 2024.
2. H. Xu and et al. Spacel: deep learning-based characterization of spatial transcriptome architectures. *Nature Communications*, 14:7603, 2023.
