# Supplementary material for: Improving cell-type composition inference in spatial transcriptomics with SpaDAMA
Source: PLoS Comput Biol. 2025 Aug 21;21(8):e1013354. doi: 10.1371/journal.pcbi.1013354 (PMC12393736; doi:10.1371/journal.pcbi.1013354)
Supplement: S1 Fig — Metrics include Pearson correlation coefficient (PCC), Structural Similarity Index Measure (SSIM), Root Mean Square Error (RMSE), and Jensen-Shannon (JS) divergence. (PDF) [file pcbi.1013354.s002.pdf]

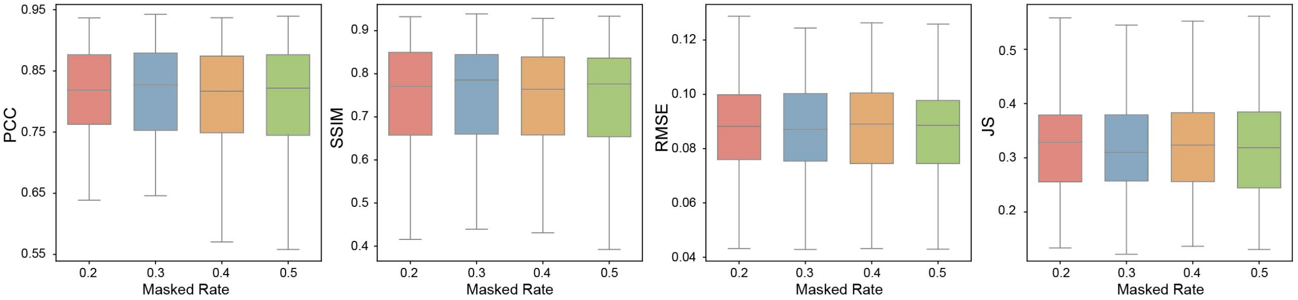

S1 Fig. We evaluated the PCC, SSIM, RMSE, and JS values of SpaDAMA at different mask rates ρ in the 32 simulated datasets.
