# Supplementary material for: Improving cell-type composition inference in spatial transcriptomics with SpaDAMA
Source: PLoS Comput Biol. 2025 Aug 21;21(8):e1013354. doi: 10.1371/journal.pcbi.1013354 (PMC12393736; doi:10.1371/journal.pcbi.1013354)
Supplement: S2 Fig — Datasets 1 to 3 are evaluated with Pearson correlation coefficient (PCC), and Dataset 4 is evaluated with Area Under the Curve (AUC). (PDF) [file pcbi.1013354.s003.pdf]

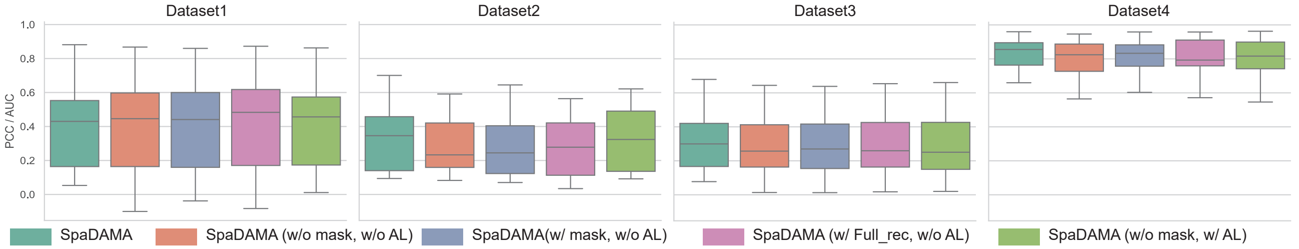

S2 Fig. Ablation study results on real spatial transcriptomics datasets. Datasets 1 to 3 are evaluated using Pearson correlation coe icient (PCC), while Dataset 4 uses Area Under the Curve (AUC) as the metric.
