# Supplementary material for: Improving cell-type composition inference in spatial transcriptomics with SpaDAMA
Source: PLoS Comput Biol. 2025 Aug 21;21(8):e1013354. doi: 10.1371/journal.pcbi.1013354 (PMC12393736; doi:10.1371/journal.pcbi.1013354)
Supplement: S3 Fig — (A) UMAP clustering plot showing distinct scRNA-seq cell type clusters. (B) Marker genes selected for each cell type, highlighting characteristic expression patterns. (PDF) [file pcbi.1013354.s004.pdf]

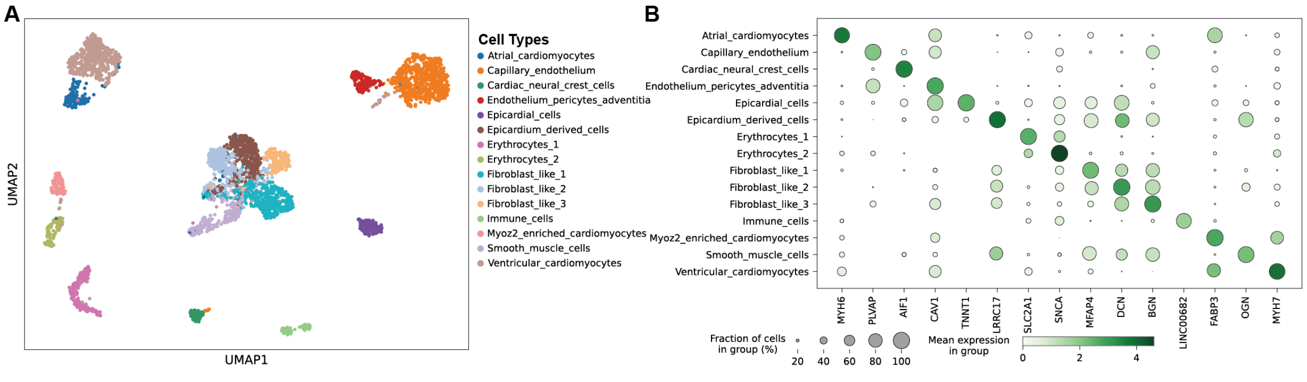

S3 Fig. SpaDAMA analyzes cell types in the Human Developing Heart (HDH) dataset. (**A**) Clustering results of scRNA-seq data based on cell types from the same tissue. The UMAP clustering plot reveals distinct clusters corresponding to different c ell t ypes, w ith s ignificant di fferences in gene expression profiles. S imilar c ell s ubtypes (e.g., t he t hree fi broblast su bgroups) cl uster to gether, su ggesting po tential fu nctional or developmental relationships, possibly reflecting s hared b iological p rocesses o r s ignaling p athways. (**B**) M arker g enes s elected f or e ach c ell t ype. T he m arker genes show the highest expression levels and relative abundance within their respective cell populations, further supporting the distinct gene expression characteristics between different c ell types.
