# Supplementary material for: Improving cell-type composition inference in spatial transcriptomics with SpaDAMA
Source: PLoS Comput Biol. 2025 Aug 21;21(8):e1013354. doi: 10.1371/journal.pcbi.1013354 (PMC12393736; doi:10.1371/journal.pcbi.1013354)
Supplement: S5 Fig — Includes total loss, prediction loss, reconstruction loss on masked input, classification loss, and discrimination losses under correct and label-inverted conditions. (PDF) [file pcbi.1013354.s006.pdf]

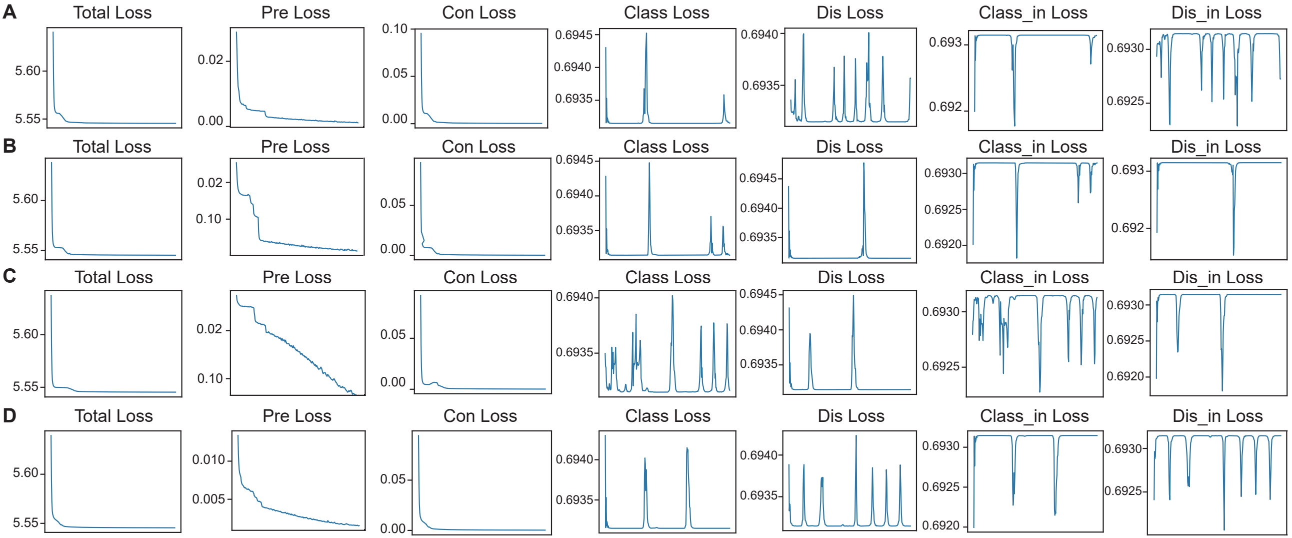

S5 Fig. SpaDAMA loss curves across four real datasets. *Total Loss* represents the overall loss function. *Pre Loss* denotes the prediction loss, while *Con Loss* corresponds to the reconstruction loss on the masked input. *Class Loss* and *Dis Loss* indicate the classification and discrimination losses under correct labels, respectively. *Class_in Loss* and *Dis_in Loss* represent the classification and discrimination losses under label-inverted conditions, respectively. (**A**) Human Developing Heart (HDH) dataset. (**B**) Murine Lymph Node (MLN) dataset. (**C**) Zebrafish Embryo (ZE) dataset. (**D**) Human Pancreatic Ductal Adenocarcinoma (PDAC) dataset.
