# Supplementary material for: Improving cell-type composition inference in spatial transcriptomics with SpaDAMA
Source: PLoS Comput Biol. 2025 Aug 21;21(8):e1013354. doi: 10.1371/journal.pcbi.1013354 (PMC12393736; doi:10.1371/journal.pcbi.1013354)
Supplement: S6 Fig — (PDF) [file pcbi.1013354.s007.pdf]

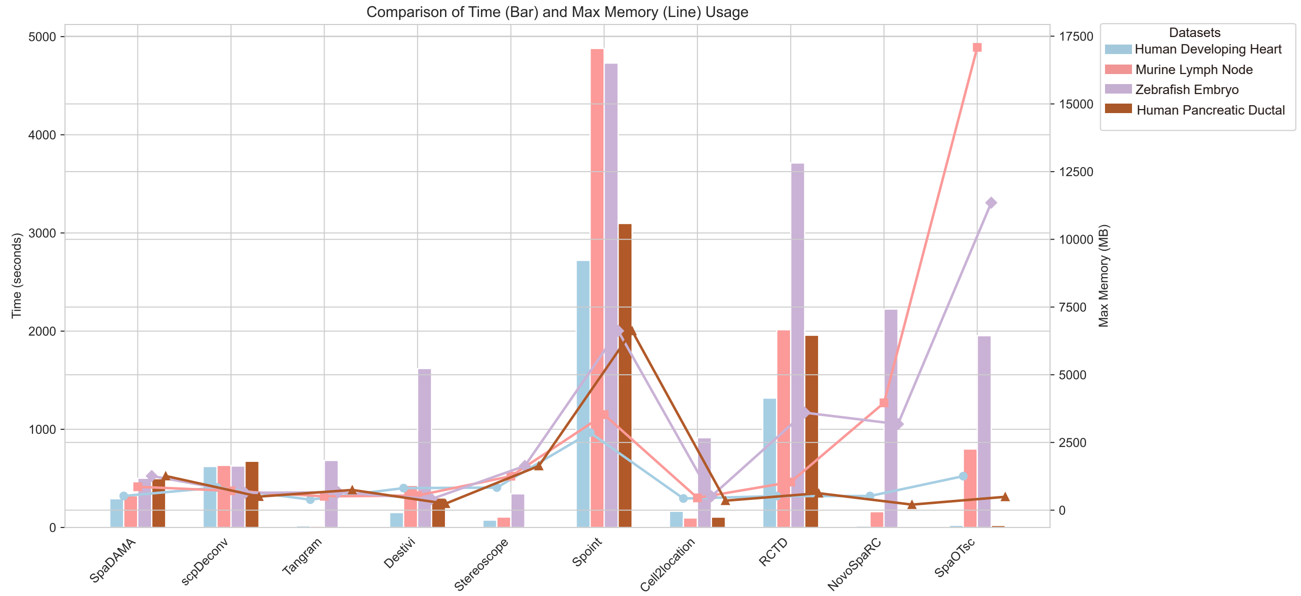

S6 Fig. Runtime and memory usage comparison of nine deconvolution methods across four real-world spatial transcriptomics datasets. Bar plots represent the runtime (in seconds) of each method on four datasets of varying sizes, while line plots indicate the corresponding peak memory usage (in megabytes). When the dataset size is small (e.g., fewer than 2000 spatial spots), methods such as Tangram, novoSpaRc, and SpaOTsc demonstrate notably fast runtimes—often completing within a few seconds. Particularly, novoSpaRc and SpaOTsc tend to finish a lmost i nstantly o n d atasets with fewer than 1000 spots, making their execution times visually compressed in the bar plot. This visualization highlights both the computational efficiency and memory demands of each method under varying data scales.
