# Supplementary material for: Improving cell-type composition inference in spatial transcriptomics with SpaDAMA
Source: PLoS Comput Biol. 2025 Aug 21;21(8):e1013354. doi: 10.1371/journal.pcbi.1013354 (PMC12393736; doi:10.1371/journal.pcbi.1013354)
Supplement: S1 Table — (PDF) [file pcbi.1013354.s008.pdf]

S1 Table. Cell type distributions by tissue region in PDAC.

Region	Cell Types
Duct epithelium	Ductal_CRISP3_high-centroacinar_like,Ductal_MHC_Class_II,Tuft_cells,Ductal_terminal_ductal_like
Cancer	Cancer_clone_A,Cancer_clone_B
Pancreatic	Acinar_cells,Endocrine_cells,pDCs,T_cells_&_NK_cells,RBCs,mDCs_A,mDCs_B
Stroma	Fibroblasts,Macrophages_A,Macrophages_B ,Mast_cells,Monocytes,Endothelial_cells
