# Supplementary material for: Improving cell-type composition inference in spatial transcriptomics with SpaDAMA
Source: PLoS Comput Biol. 2025 Aug 21;21(8):e1013354. doi: 10.1371/journal.pcbi.1013354 (PMC12393736; doi:10.1371/journal.pcbi.1013354)
Supplement: S2 Table — Includes tissue type, data types (scRNA-seq and ST), cell and spot counts, gene counts, and dropout rates for source and preprocessed data. (PDF) [file pcbi.1013354.s009.pdf]

S2 Table. Detailed information of each dataset. The scRNA-seq data in Dataset1 to Dataset32 are real data, and the matching ST data are simulated, while the scRNA-seq and ST data in Dataset33 to Dataset36 are both real data.

Datasets	Tissue	data_type		source_data				preproce_data					
		SC	ST	Number of Cells/Spots		Number of Genes		Number of Cells/Spots		Number of Genes		Droupout rate	
				SC (Cells)	ST (Spots)	SC	ST	SC (Cells)	ST (Spots)	SC	ST	SC	ST
Dataset1	Human-Brain[1]	Real	Simulated	10000	1000	33691	46732	10000	1000	4456	4456	81.29%	61.01%
Dataset2	Mouse-Brain[1]	Real	Simulated	10000	1000	43324	24683	10000	1000	4481	4481	61.05%	76.30%
Dataset3	Human-Liver[2]	Real	Simulated	3821	1000	18328	19850	3821	1000	1186	1186	90.06%	94.83%
Dataset4	Human-Liver[3]	Real	Simulated	6948	1000	20007	26160	6948	1000	1171	1171	92.08%	86.78%
Dataset5	Human-Lung[4]	Real	Simulated	10000	1000	25734	38150	10000	1000	2303	2303	88.12%	95.32%
Dataset6	Human-Lung[5]	Real	Simulated	10000	1000	25734	26828	10000	1000	1639	1639	84.16%	89.17%
Dataset7	Human-Lung[6]	Real	Simulated	10000	1000	22066	25199	10000	1000	2831	2831	88.54%	90.01%
Dataset8	Human-Kidney[7]	Real	Simulated	10000	1000	27345	31489	10000	1000	1531	1531	90.28%	95.15%
Dataset9	Mouse-Kidney[8]	Real	Simulated	10000	1000	24965	29244	10000	1000	1120	1120	96.76%	95.51%
Dataset10	Human-Heart[9]	Real	Simulated	10000	1000	17926	29484	10000	1000	1426	1426	82.13%	95.66%
Dataset11	Human-Heart[9]	Real	Simulated	10000	1000	17926	31580	10000	1000	1426	1426	82.75%	92.18%
Dataset12	Human-Pancreas[10]	Real	Simulated	2282	1000	21198	17499	2282	1000	1077	1077	79.84%	85.17%
Dataset13	Human-Pancreas[10]	Real	Simulated	1040	1000	21625	17499	1040	1000	1427	1427	64.85%	85.48%
Dataset14	Human-Pancreas[11]	Real	Simulated	943	1000	21625	21198	943	1000	1135	1135	66.08%	45.05%
Dataset15	Mouse-Pancreas[10]	Real	Simulated	1382	1000	19745	14860	1382	1000	1504	1504	67.44%	84.52%
Dataset16	Mouse-Trachea[12]	Real	Simulated	6937	1000	27084	18388	6937	1000	1034	1034	87.83%	87.74%
Dataset17	Human-Brain[1]	Real	Simulated	10000	1000	46732	33691	10000	1000	4942	4942	85.99%	79.82%
Dataset18	Mouse-Brain[1]	Real	Simulated	9999	1000	24683	43324	9999	1000	3227	3227	78.03%	37.39%
Dataset19	Human-Liver[13]	Real	Simulated	10000	1000	19850	18328	10000	1000	1183	1183	95.88%	87.03%
Dataset20	Human-Liver[14]	Real	Simulated	8785	1000	26160	20007	8785	1000	1101	1101	90.33%	90.97%
Dataset21	Human-Lung[15]	Real	Simulated	10000	1000	38150	25734	10000	1000	1813	1813	96.07%	55.29%
Dataset22	Human-Lung[16]	Real	Simulated	10000	1000	26828	25734	10000	1000	1502	1502	90.63%	44.66%
Dataset23	Human-Lung[17]	Real	Simulated	10000	1000	25199	22066	10000	1000	2535	2535	92.00%	86.07%
Dataset24	Human-Kidney[18]	Real	Simulated	10000	1000	31489	27345	10000	1000	1218	1218	95.91%	87.56%
Dataset25	Mouse-Kidney[19]	Real	Simulated	10000	1000	29244	24965	10000	1000	885	885	96.63%	94.99%
Dataset26	Human-Heart[20]	Real	Simulated	10000	1000	29484	17926	10000	1000	1077	1077	95.42%	75.15%
Dataset27	Human-Heart[21]	Real	Simulated	10000	1000	31580	17926	10000	1000	1358	1358	93.58%	77.14%
Dataset28	Human-Pancreas[11]	Real	Simulated	7944	1000	17499	21198	7944	1000	1148	1148	87.47%	41.59%
Dataset29	Human-Pancreas[1]	Real	Simulated	8494	1000	17499	21625	8494	1000	1559	1559	87.80%	32.99%
Dataset30	Human-Pancreas[1]	Real	Simulated	2282	1000	21198	21625	2282	1000	1080	1080	81.47%	38.31%
Dataset31	Mouse-Pancreas[22]	Real	Simulated	1827	1000	14860	19745	1827	1000	1143	1143	87.02%	24.93%
Dataset32	Mouse-Trachea[23]	Real	Simulated	7128	1000	18388	27084	7128	1000	1006	1006	88.17%	82.86%
Dataset33	Human Developing Heart[24]	Real	Real	3777	210	15323	38936	3777	209	2373	2373	81.00%	94.01%
Dataset34	Murine Lymph Node[25]	Real	Real	14989	1092	12854	13948	14989	1092	1870	1870	83.41%	61.09%
Dataset35	Zebrafish Embryo[26]	Real	Real	9903	3048	23986	23110	9903	3048	2396	2396	0.00%	0.00%
Dataset36	Human Pancreatic Ductal[27]	Real	Real	1926	426	14121	14121	1926	426	1575	1575	0.00%	0.00%

References

1. B. Li, W. Zhang, and et al. Benchmarking spatial and single-cell transcriptomics integration methods for transcript distribution prediction and cell type deconvolution. *Nature Methods*, 19:662–670, 2022.
2. L. et al. Ma. Tumor cell biodiversity drives microenvironmental reprogramming in liver cancer. *Cancer Cell*, 36(4):418–430.e6, 2019.
3. S. A. et al. MacParland. Single cell rna sequencing of human liver reveals distinct intrahepatic macrophage populations. *Nature Communications*, 9(1):4383, 2018.
4. F. et al. Fan. Elevated mast cell abundance is associated with enrichment of ccr2+ cytotoxic t cells and favorable prognosis in lung adenocarcinoma. *Cancer Research*, 83(16):2690–2703, Aug 2023.
5. Z. et al. Wang. Single-cell transcriptomic analysis reveals an immunosuppressive network between postn cafs and ackr1 ecs in tki-resistant lung cancer. *Cancer Genomics & Proteomics*, 21(1):65–78, Jan–Feb 2024.
6. X. et al. Jin. Dissection of the cell communication interactions in lung adenocarcinoma identified a prognostic model with immunotherapy efficacy assessment and a potential therapeutic candidate gene itgb1. *Heliyon*, 10(17):e36599, Sep 2024.
7. B. B. et al. Lake. A single-nucleus rna-sequencing pipeline to decipher the molecular anatomy and pathophysiology of human kidneys. *Nature Communications*, 10(1):2832, Jun 2019.
8. J. et al. Cao. Joint profiling of chromatin accessibility and gene expression in thousands of single cells. *Science*, 361(6409):1380–1385, Sep 2018.
9. C. B. et al. Sim. Sex-specific control of human heart maturation by the progesterone receptor. *Circulation*, 143(16):1614–1628, Apr 2021.
10. M. et al. Baron. A single-cell transcriptomic map of the human and mouse pancreas reveals inter- and intra-cell population structure. *Cell Systems*, 3(4):346–360.e4, Oct 2016.
11. M. et al. Enge. Single-cell analysis of human pancreas reveals transcriptional signatures of aging and somatic mutation patterns. *Cell*, 171(2):321–330.e14, Oct 2017.
12. D. T. et al. Montoro. A revised airway epithelial hierarchy includes cftr-expressing ionocytes. *Nature*, 560(7718):319–324, Aug 2018.
13. A. et al. Sharma. Onco-fetal reprogramming of endothelial cells drives immunosuppressive macrophages in hepatocellular carcinoma. *Cell*, 183(2):377–394.e21, Oct 2020.
14. N. et al. Aizarani. A human liver cell atlas reveals heterogeneity and epithelial progenitors. *Nature*, 572(7768):199–204, Aug 2019.
15. R. et al. Zilionis. Single-cell transcriptomics of human and mouse lung cancers reveals conserved myeloid populations across individuals and species. *Immunity*, 50(5):1317–1334.e10, May 2019.
16. N. et al. Kim. Single-cell rna sequencing demonstrates the molecular and cellular reprogramming of metastatic lung adenocarcinoma. *Nature Communications*, 11(1):2285, May 2020.
17. Synapse. Dataset: syn21041850, 2024. Accessed: 2024-12-27.
18. Y. et al. Muto. Defining cellular complexity in human autosomal dominant polycystic kidney disease by multimodal single cell analysis. *Nature Communications*, 13(1):6497, Oct 2022.
19. Y. et al. Muto. Single cell transcriptional and chromatin accessibility profiling redefine cellular heterogeneity in the adult human kidney. *Nature Communications*, 12(1):2190, Apr 2021.
20. N. R. et al. Tucker. Transcriptional and cellular diversity of the human heart. *Nature*, 2024. In press.
21. A. et al. Daoud. Integrative single-cell analysis of cardiac and pulmonary sarcoidosis using publicly available cardiac and bronchoalveolar lavage fluid sequencing datasets. *Frontiers in Cardiovascular Medicine*, 10:101227818, 2023.
22. Tabula Muris Consortium et al. Single-cell transcriptomics of 20 mouse organs creates a tabula muris. *Nature*, 562(7727):367–372, Oct 2018.
23. L. W. et al. Plasschaert. A single-cell atlas of the airway epithelium reveals the cftr-rich pulmonary ionocyte. *Nature*, 560(7718):377–381, Aug 2018.
24. M. Asp, S. Giacomello, and et al. A spatiotemporal organ-wide gene expression and cell atlas of the developing human heart. *Cell*, 179(7):1647–1660.e19, 2019.
25. R.and et al. Lopez. Destvi identifies continuums of cell types in spatial transcriptomics data. *Nature Biotechnology*, 40:1360–1369, 2022.
26. H. Li, J. Zhou, Z. Li, et al. A Comprehensive Benchmarking with Practical Guidelines for Cellular Deconvolution of Spatial Transcriptomics. *Nature Communications*, 14:1548, 2023.
27. Y. Ma and X. Zhou. Spatially Informed Cell-Type Deconvolution for Spatial Transcriptomics. *Nature Biotechnology*, 40(10):1349–1359, 2022.
