# Supplementary material for: Improving cell-type composition inference in spatial transcriptomics with SpaDAMA
Source: PLoS Comput Biol. 2025 Aug 21;21(8):e1013354. doi: 10.1371/journal.pcbi.1013354 (PMC12393736; doi:10.1371/journal.pcbi.1013354)
Supplement: S3 Table — Comparisons among baseline models with and without masking and adversarial learning, and full SpaDAMA model. (PDF) [file pcbi.1013354.s010.pdf]

S3 Table. Ablation experiments on the 32 simulated datasets in terms of the AS metric. AL: Adversarial Learning. mask: Reconstruction loss computed only for the masked points. Full rec: Full reconstruction loss. SpaDAMA (w/o mask, w/o AL): Baseline model with only the encoder and predictor, no mask; SpaDAMA (w/ mask, w/o AL): Baseline model with only the encoder and predictor, with mask, loss computed only for masked points; SpaDAMA (w/o mask, w/ AL): Baseline model with all modules, no mask, adversarial learning added; SpaDAMA (w/Full rec, w/o AL): Baseline model with only the encoder and predictor, with mask, full reconstruction loss.

**AS(↑)**	Dataset1	Dataset2	Dataset3	Dataset4	Dataset5	Dataset6	dataset7	dataset8
SpaDAMA (w/o mask, w/o AL)	0.638±0.054	0.563±0.042	0.538±0.082	0.363±0.021	0.425±0.018	0.438±0.052	0.513±0.036	0.413±0.036
SpaDAMA (w/ mask,w/o AL)	0.425±0.049	0.813±0.017	0.313±0.051	0.425±0.014	0.475±0.031	0.625±0.008	0.425±0.024	0.413±0.009
SpaDAMA (w/o mask, w/ AL)	0.700±0.008	0.363±0.021	0.725±0.018	0.800±0.007	0.525±0.011	0.650±0.035	0.638±0.026	0.800±0.012
SpaDAMA (w/ Full_rec, w/o AL)	0.513±0.011	0.375±0.018	0.625±0.034	0.550±0.065	0.575±0.058	0.375±0.016	0.463±0.041	0.475±0.048
SpaDAMA	0.725±0.068	0.888±0.007	0.800±0.015	0.863±0.024	0.998±0.001	0.913±0.011	0.963±0.006	0.900±0.015

**AS(↑)**	Dataset9	Dataset10	Dataset11	Dataset12	Dataset13	Dataset14	Dataset15	Dataset16
SpaDAMA (w/o mask, w/o AL)	0.550±0.022	0.425±0.028	0.400±0.008	0.450±0.022	0.400±0.012	0.400±0.062	0.363±0.027	0.513±0.051
SpaDAMA (w/ mask, w/o AL)	0.625±0.016	0.375±0.038	0.388±0.012	0.588±0.037	0.763±0.009	0.650±0.007	0.425±0.001	0.400±0.028
SpaDAMA (w/o mask, w/ AL)	0.700±0.008	0.825±0.004	0.775±0.011	0.663±0.052	0.638±0.027	0.588±0.006	0.750±0.028	0.563±0.004
SpaDAMA (w/ Full_rec, w/o AL)	0.325±0.001	0.425±0.011	0.463±0.037	0.475±0.084	0.325±0.004	0.363±0.012	0.550±0.035	0.588±0.021
SpaDAMA	0.800±0.035	0.950±0.005	0.975±0.003	0.825±0.038	0.875±0.008	1.000±0.000	0.913±0.004	0.938±0.006

**AS(↑)**	Dataset17	Dataset18	Dataset19	Dataset20	Dataset21	Dataset22	Dataset23	Dataset24
SpaDAMA (w/o mask, w/o AL)	0.425±0.024	0.750±0.032	0.375±0.018	0.363±0.036	0.588±0.007	0.525±0.041	0.450±0.015	0.425±0.041
SpaDAMA (w/ mask,w/o AL)	0.488±0.074	0.400±0.032	0.575±0.091	0.325±0.023	0.650±0.002	0.488±0.024	0.525±0.034	0.888±0.021
SpaDAMA (w/o mask, w/ AL)	0.638±0.012	0.438±0.036	0.650±0.055	0.775±0.003	0.650±0.045	0.763±0.026	0.500±0.012	0.338±0.002
SpaDAMA (w/ Full_rec,w/o AL)	0.550±0.052	0.488±0.046	0.650±0.062	0.638±0.037	0.238±0.006	0.400±0.073	0.525±0.034	0.563±0.037
SpaDAMA	0.900±0.015	0.925±0.003	0.750±0.138	0.900±0.028	0.875±0.014	0.825±0.038	0.998±0.001	0.788±0.022

**AS(↑)**	Dataset25	Dataset26	Dataset27	Dataset28	Dataset29	Dataset30	Dataset31	Dataset32
SpaDAMA (w/o mask, w/o AL)	0.463±0.026	0.413±0.057	0.488±0.002	0.388±0.022	0.613±0.036	0.550±0.047	0.588±0.052	0.538±0.056
SpaDAMA (w/ mask,w/o AL)	0.738±0.017	0.300±0.008	0.513±0.022	0.500±0.028	0.800±0.028	0.638±0.029	0.425±0.043	0.313±0.011
SpaDAMA (w/o mask, w/ AL)	0.613±0.026	0.763±0.027	0.750±0.052	0.888±0.011	0.488±0.022	0.475±0.008	0.663±0.012	0.525±0.043
SpaDAMA (w/ Full_rec,w/o AL)	0.275±0.009	0.613±0.042	0.325±0.004	0.450±0.042	0.400±0.022	0.513±0.012	0.363±0.021	0.738±0.021
SpaDAMA	0.913±0.002	0.913±0.011	0.925±0.004	0.775±0.069	0.700±0.105	0.825±0.014	0.963±0.002	0.888±0.037
