# Supplementary material for: Improving cell-type composition inference in spatial transcriptomics with SpaDAMA
Source: PLoS Comput Biol. 2025 Aug 21;21(8):e1013354. doi: 10.1371/journal.pcbi.1013354 (PMC12393736; doi:10.1371/journal.pcbi.1013354)
Supplement: S4 Table — (PDF) [file pcbi.1013354.s011.pdf]

S4 Table. Recommended parameter settings for simulating the number of cells and cell types per spot across different ST technologies.

**Platform**	μ_c (cells/spot)	δ_c	μ_t (cell types/spot)	δ_t	**Notes**
Visium	6–10	2–4	3–5	1.5–2.5	Medium resolution; multiple cells per spot
Slide-seqV2	1–3	1	1–2	0.5	Near single-cell resolution
MERFISH	1	0.5	1	0	True single-cell resolution
HDST	1–2	0.5–1	1–2	0.5	High resolution, small tissue structures
