# Supplementary material for: Improving cell-type composition inference in spatial transcriptomics with SpaDAMA
Source: PLoS Comput Biol. 2025 Aug 21;21(8):e1013354. doi: 10.1371/journal.pcbi.1013354 (PMC12393736; doi:10.1371/journal.pcbi.1013354)
Supplement: S4 Fig — (PDF) [file pcbi.1013354.s005.pdf]

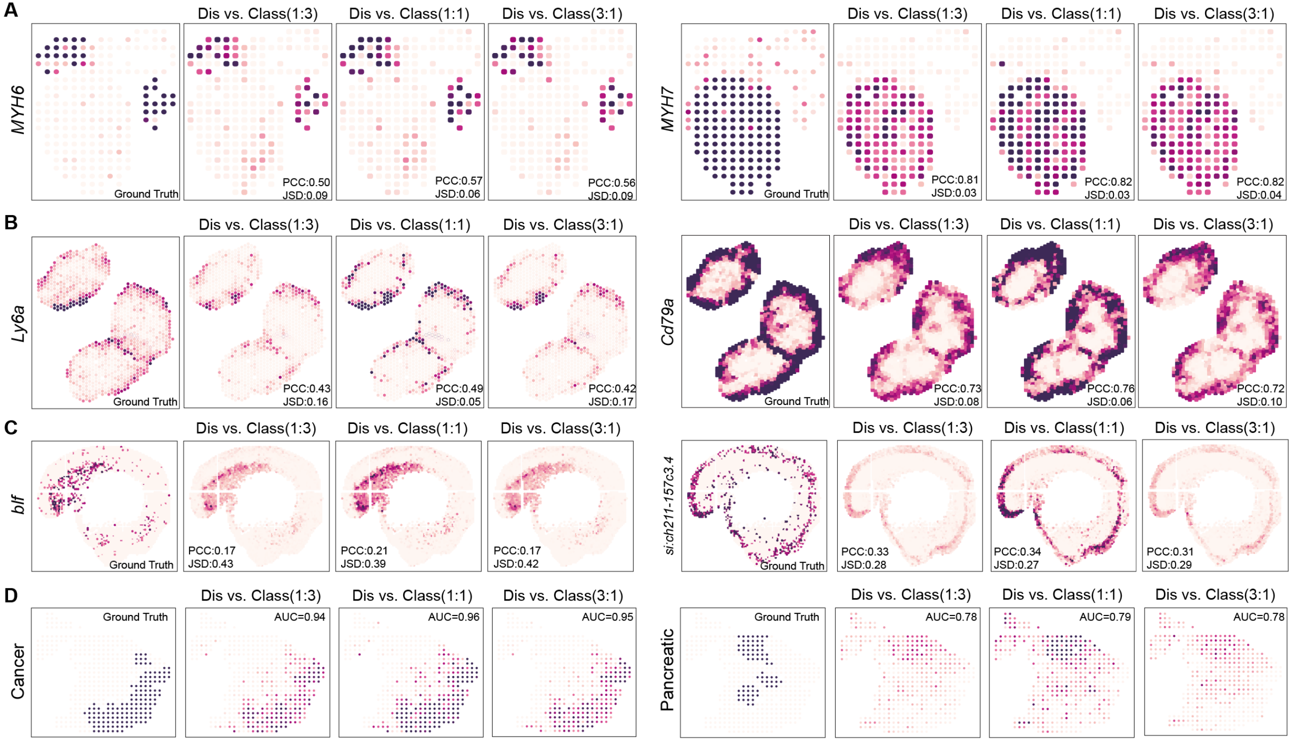

S4 Fig. Performance of SpaDAMA under different latent feature allocation ratios between the classifier and discriminator across four real spatial transcriptomics datasets. (**A**) Human Developing Heart (HDH) dataset. (**B**) Murine Lymph Node (MLN) dataset. (**C**) Zebrafish Embryo (ZE) dataset. (**D**) Human Pancreatic Ductal Adenocarcinoma (PDAC) dataset.
